# Adsorption of Aldehyde-Functional Diblock Copolymer Spheres onto Surface-Grafted Polymer Brushes via Dynamic Covalent Chemistry Enables Friction Modification

**DOI:** 10.1021/acs.chemmater.3c01227

**Published:** 2023-07-19

**Authors:** Edwin C. Johnson, Spyridon Varlas, Oleta Norvilaite, Thomas J. Neal, Emma E. Brotherton, George Sanderson, Graham J. Leggett, Steven P. Armes

**Affiliations:** † Department of Chemistry, University of Sheffield, Dainton Building, Brook Hill, Sheffield S3 7HF, U.K.; ‡ GEO Specialty Chemicals, Hythe, Southampton SO45 3ZG, U.K.

## Abstract

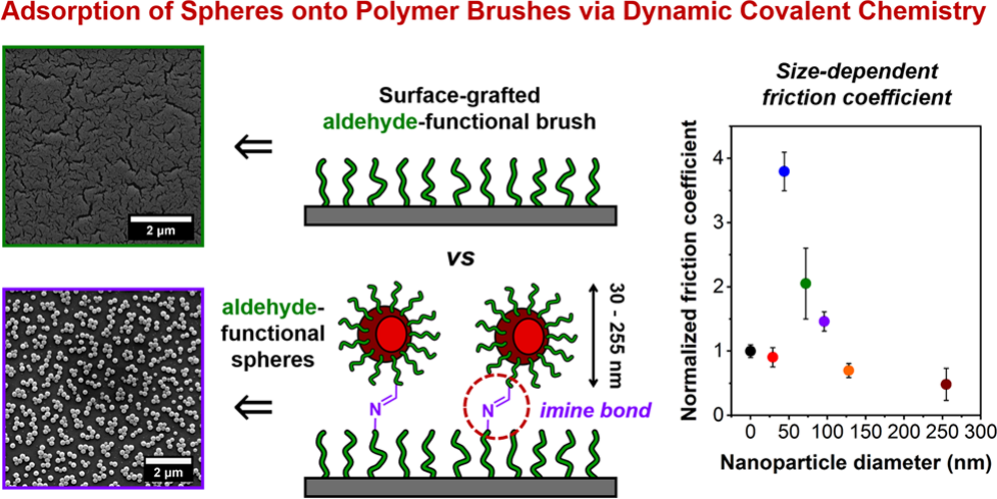

Dynamic covalent chemistry has been exploited to prepare numerous examples of adaptable polymeric materials that exhibit unique properties. Herein, the chemical adsorption of aldehyde-functional diblock copolymer spherical nanoparticles onto amine-functionalized surface-grafted polymer brushes via dynamic Schiff base chemistry is demonstrated. Initially, a series of *cis*-diol-functional sterically-stabilized spheres of 30–250 nm diameter were prepared via reversible addition–fragmentation chain transfer (RAFT) aqueous dispersion polymerization. The pendent *cis*-diol groups within the steric stabilizer chains of these precursor nanoparticles were then oxidized using sodium periodate to produce the corresponding aldehyde-functional spheres. Similarly, hydrophilic *cis*-diol-functionalized methacrylic brushes grafted from a planar silicon surface using activators regenerated by electron transfer atom transfer radical polymerization (ARGET ATRP) were selectively oxidized to generate the corresponding aldehyde-functional brushes. Ellipsometry and X-ray photoelectron spectroscopy were used to confirm brush oxidation, while scanning electron microscopy studies demonstrated that the nanoparticles did not adsorb onto a *cis*-diol-functional precursor brush. Subsequently, the aldehyde-functional brushes were treated with excess small-molecule diamine, and the resulting imine linkages were converted into secondary amine bonds via reductive amination. The resulting primary amine-functionalized brushes formed multiple dynamic imine bonds with the aldehyde-functional diblock copolymer spheres, leading to a mean surface coverage of approximately 0.33 on the upper brush layer surface, regardless of the nanoparticle size. Friction force microscopy studies of the resulting nanoparticle-decorated brushes enabled calculation of friction coefficients, which were compared to that measured for the bare aldehyde-functional brush. Friction coefficients were reasonably consistent across all surfaces except when particle size was comparable to the size of the probe tip. In this case, differences were ascribed to an increase in contact area between the tip and the brush-nanoparticle layer. This new model system enhances our understanding of nanoparticle adsorption onto hydrophilic brush layers.

## Introduction

Dynamic covalent chemistry (DCC) involves the formation of dynamic covalent bonds (DCBs) under equilibrium conditions.^1−5^ DCC can be used to design complex multi-component materials or to generate combinatorial libraries.^3^ Thus far, it has been successfully exploited for (bio)synthetic templating,^3,6−8^ mechanochemistry,^9,10^ construction of metal–organic frameworks,^11^ as well as polymeric and colloidal materials engineering.^5^

In the context of polymer science, DCC has enabled the development of self-healing polymers and mechanically/macroscopically-responsive materials with adaptable properties.^12−17^ It has also been utilized to design stimulus-responsive polymers, either through modification of their characteristics or by responding directly to the formation of DCBs, such as imine bonds,^18,19^ acylhydrazone bonds,^20^ Diels–Alder chemistry,^21^ Se–N bonds,^22^ boronic acid/*cis*-diol binding,^23−27^ etc. Of particular relevance to the present study are imine bonds, which are formed when reacting an aldehyde with a primary amine. Such Schiff base chemistry has been used to generate covalent adaptable networks (CANs)^28,29^ and hydrogels^5^ for potential biomedical applications,^30^ while it has also been exploited for conjugation of oligonucleotides to aldehyde-functional polymer brushes, which were prepared via partial oxidation of a poly(2-hydroxyethyl methacrylate) (PHEMA) precursor brush.^31^

Recently, we reported a new hydrophilic aldehyde-functional polymer (PAGEO5MA).^32−34^ This polymer can either be directly synthesized from the corresponding methacrylic monomer (AGEO5MA) or it can be generated via selective oxidation of a *cis*-diol-functional PGEO5MA precursor using sodium periodate (NaIO_4_).^32^ PAGEO5MA-based diblock copolymer spheres or vesicles can be reacted with various amino acids (e.g., glycine or l-histidine) via Schiff base chemistry followed by reductive amination.^33,35^ PAGEO5MA-based diblock copolymer worms form soft, free-standing gels that exhibit strong mucoadhesion,^34^ while PAGEO5MA brushes grafted from a planar surface can be reacted with model globular proteins, such as bovine serum albumin (BSA).^36^ This involves the formation of multiple imine bonds between the aldehyde groups on the brush chains and the primary amine groups located at the surface of the protein. Each individual imine bond is relatively weak and prone to hydrolysis, but a sufficient number of these bonds remain intact at any given time to ensure that the protein remains conjugated to the polymer brush.

The design of well-defined nanoparticle-brush hybrid surfaces enables the construction of complex interfacial structures and multicomponent materials with tailored properties. This offers potential applications ranging from energy storage and plasmonics to drug release and friction modification.^37−39^ For example, Huck and co-workers examined the adsorption of gold nanoparticles within or onto polymer brushes.^39−42^ In this case, nanoparticle adsorption occurred via non-covalent electrostatic interactions, rather than by DCC.

**Scheme 1:**
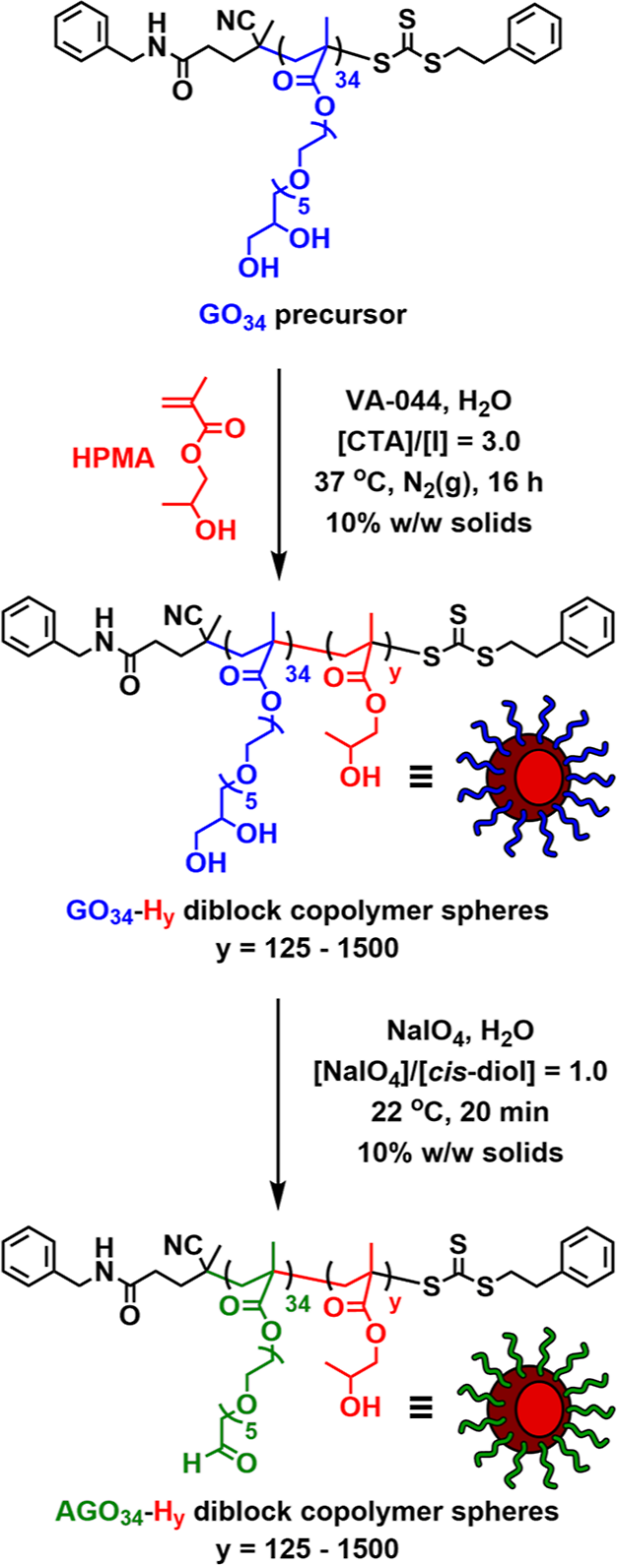
Schematic Illustration of the Synthesis of *cis*-Diol-Functional GO_34_-H_*y*_ Diblock Copolymer Spheres (where *y* = 125–1500) via RAFT Aqueous Dispersion Polymerization of HPMA at 10% w/w Solids Using a GO_34_ Precursor, and Their Subsequent Selective Oxidation Using NaIO_4_ for the Preparation of Aldehyde-Functional AGO_34_-H_*y*_ Diblock Copolymer Spheres at 10% w/w Solids

Herein, we report the adsorption of aldehyde-functional diblock copolymer spherical nanoparticles of varying size onto amine-functionalized surface-grafted polymer brushes via DCC to generate a library of nanoparticle-brush planar surfaces with tunable friction coefficients. More specifically, a PGEO5MA_34_ precursor was chain-extended with 2-hydroxypropyl methacrylate (HPMA) to generate a series of kinetically-trapped diblock copolymer spheres via reversible addition–fragmentation chain transfer (RAFT) aqueous dispersion polymerization (Scheme 1). These PGEO5MA_34_-PHPMA_*y*_ (denoted GO_34_-H_*y*_) diblock copolymer nanoparticles were analyzed by dynamic light scattering (DLS), small-angle X-ray scattering (SAXS), and electron microscopy. Their pendent *cis*-diol groups were then selectively oxidized using a stoichiometric amount of NaIO_4_ under mild conditions with no loss of colloidal stability. The resulting aldehyde-functional PAGEO5MA_34_-PHPMA_*y*_ (denoted AGO_34_-H_*y*_) nanoparticles were then exposed to a series of hydrophilic PAGEO5MA brushes, which had been previously reacted with a small-molecule diamine. Dynamic imine bond formation between the nanoparticles and the primary amine-functional brushes led to their chemical adsorption. Subsequently, friction force microscopy was used to examine the relationship between nanoparticle diameter, surface topography, and measured friction coefficient.

## Results and Discussion

A PGEO5MA_34_ (or GO_34_) precursor was first synthesized via RAFT solution polymerization of GEO5MA in ethanol at 70 °C using a non-ionic trithiocarbonate chain transfer agent, following a similar experimental protocol to that previously reported by our group (Scheme S2).^32,33^ A final GEO5MA conversion of 86% was achieved after 180 min, as determined by ^1^H NMR analysis in CD_3_OD. End-group analysis of the purified PGEO5MA homopolymer using the same technique indicated a mean degree of polymerization (DP) of 34 (Figure S1). Size exclusion chromatography (SEC) analysis (DMF eluent containing 10 mM LiBr) for this GO_34_ precursor revealed a narrow molecular weight distribution with an apparent number-average molecular weight (*M*_n_) of 15.8 kg mol^–1^ and a relatively low dispersity (*M*_w_/*M*_n_) of 1.16 (Figure S2). This latter parameter suggested a well-controlled RAFT polymerization.

**Figure 1:**
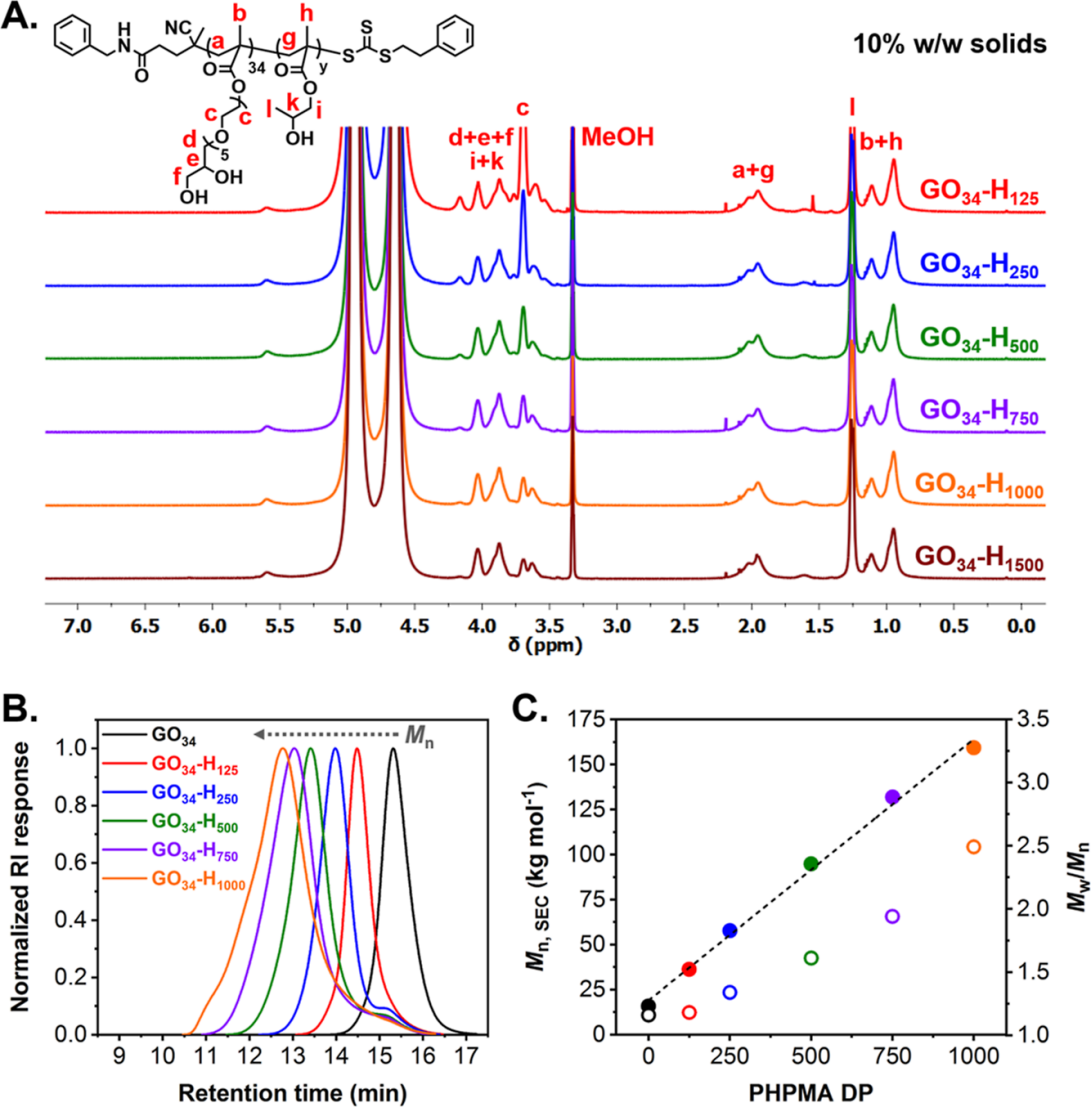
(A) Stacked ^1^H NMR spectra (CD_3_OD) recorded for a series of molecularly-dissolved GO_34_-H_*y*_ diblock copolymers (where *y* = 125–1500) prepared via RAFT aqueous dispersion polymerization of HPMA at 10% w/w solids. (B) Normalized SEC traces (refractive index detector) recorded for the GO_34_ precursor (black line), and a series of GO_34_-H_*y*_ diblock copolymers (where *y* = 125, red line; *y* = 250, blue line; *y* = 500, green line; *y* = 750, purple line; and *y* = 1000, orange line) prepared at 10% w/w solids (DMF + 10 mM LiBr eluent). (C) Evolution of *M*_n_ (filled circles) and *M*_w_/*M*_n_ (empty circles) as a function of target PHPMA DP for a series of GO_34_-H_*y*_ diblock copolymers prepared at 10% w/w solids, as determined by SEC RI analysis using a series of PMMA calibration standards.

Based on our recent experience,^33^ we anticipated that this relatively massive *cis*-diol-functional GO_34_ precursor should produce kinetically-trapped diblock copolymer spheres when conducting RAFT aqueous dispersion polymerization syntheses at a sufficiently low copolymer concentration (e.g., 10% w/w solids). Accordingly, this water-soluble GO_34_ precursor was subsequently chain-extended via RAFT aqueous dispersion polymerization of HPMA (H) at 37 °C to afford a series of GO_34_-H_*y*_ diblock copolymer spheres at 10% w/w solids (Scheme 1). The target PHPMA DP (*y*) was systematically varied from 125 to 1500 to produce nanoparticles of increasing size. Near-quantitative HPMA conversions (>99%) were achieved within 16 h in all cases, as determined by ^1^H NMR studies in CD_3_OD (Figure 1A and Table S1). SEC analysis confirmed that the GO_34_ precursor was efficiently chain-extended, with *M*_n_ values for the resulting GO_34_-H_*y*_ diblock copolymers increasing linearly when targeting higher PHPMA DPs. Relatively low dispersities (*M*_w_/*M*_n_ ≤ 1.34) were calculated for the GO_34_-H_125_ and GO_34_-H_250_ diblock copolymers, but significantly higher values were obtained when targeting longer PHPMA blocks (Figure 1B,C and Table S1). These observations are broadly consistent with previously reported data when targeting high core-forming block DPs using aqueous PISA protocols.^43^ A weak high-molecular-weight shoulder was only observed for GO_34_-H_1000_: this feature can be attributed to chain transfer to polymer or termination by recombination owing to traces of dimethacrylate impurities within the HPMA monomer.^44,45^ Unfortunately, the GO_34_-H_1500_ diblock copolymer proved to be poorly soluble in DMF and hence was unsuitable for SEC analysis.

**Figure 2:**
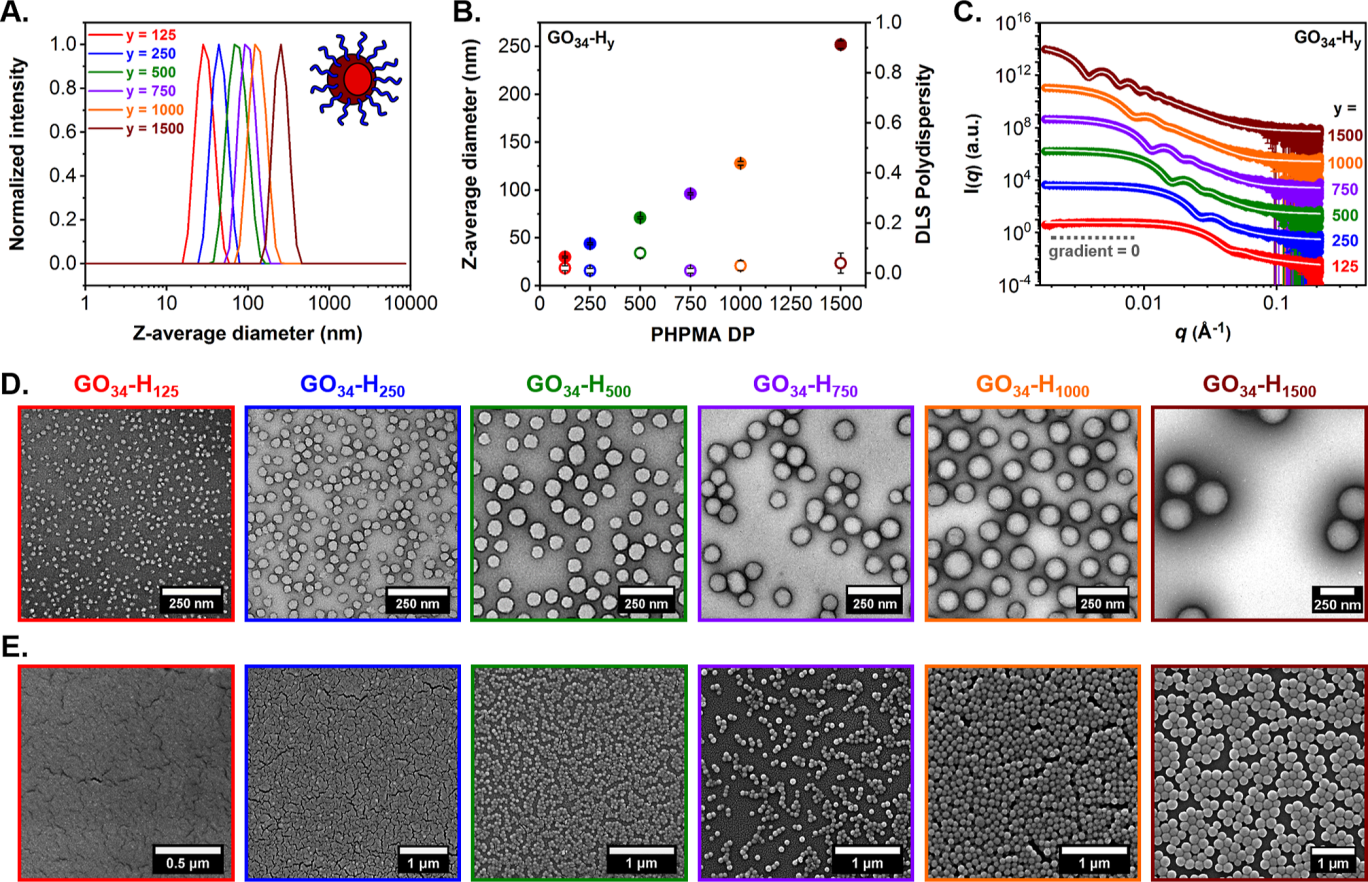
Summary of characterization data obtained for a series of *cis*-diol-functional GO_34_-H_*y*_ diblock copolymer spheres (where *y* = 125–1500) prepared via RAFT aqueous dispersion polymerization of HPMA at 10% w/w solids: (A) intensity-weighted DLS particle size distributions, (B) evolution of *z*-average diameter (filled circles) and corresponding DLS polydispersity (PD) (empty circles) with increasing target PHPMA DP, (C) SAXS patterns recorded for 1.0% w/w aqueous copolymer dispersions and corresponding data fits (white solid lines) applied to each SAXS pattern using a well-known spherical micelle model,^46^ (D) representative TEM images obtained using a uranyl formate stain, and (E) representative SEM images.

Next, the size and morphology of the GO_34_-H_*y*_ diblock copolymer nanoparticles was assessed using a series of scattering and microscopy techniques. In particular, DLS analysis confirmed narrow, monomodal particle size distributions. Targeting higher PHPMA DPs resulted in a monotonic increase in the mean hydrodynamic diameters (*D*_h_) from 30 to 250 nm with relatively low polydispersities (PD ≤ 0.08) (Figure 2A,B and Table S1). Subsequently, SAXS analysis verified these DLS data. Zero gradients were observed in the low-*q* region (where *q* is the scattering vector) of *I*(*q*) vs *q* plots, which suggested a spherical morphology in each case. Furthermore, a progressive shift in the local minimum at lower *q* was evident when targeting higher PHPMA DPs, while a satisfactory fit was obtained for each SAXS pattern using a well-documented spherical micelle model (Figure 2C).^46^ This enabled calculation of the overall volume-average particle diameters (*D*_SAXS_) for this nanoparticle series, which were consistent with the corresponding DLS data (Figure S4 and Table S1).

Morphologies for the entire series of *cis*-diol-functional GO_34_-H_*y*_ diblock copolymer nanoparticles were examined by conventional transmission electron microscopy (TEM). Well-defined, uniform spherical nanoparticles were observed in all cases (Figure 2D). Corresponding particle size distribution histograms were constructed via digital image analysis that enabled estimation of the number-average sphere diameters (*D*_TEM_) (Figure S3). As expected for particle size distributions of finite width, *D*_TEM_ values were moderately lower than *D*_SAXS_, whereas the *z*-average diameters (*D*_h_) determined by DLS were always systematically higher (Figure S4 and Table S1). Scanning electron microscopy (SEM) and atomic force microscopy (AFM) were also utilized for imaging of the dried aqueous dispersions of GO_34_-H_*y*_ diblock copolymer spheres. These complimentary techniques provided further evidence for well-defined spherical nanoparticle morphologies when examining significantly larger numbers of particles than TEM (Figures 2E and S5).

Moreover, aqueous electrophoresis studies were performed for all copolymer dispersions in a background salt concentration of 1 mM KCl at pH 6.8 (i.e., the solution pH of the deionized water used for nanoparticle syntheses, their chemical modification, and subsequent analyses) with zeta potentials ranging between −13 and −17 mV, regardless of the PHPMA DP (Figure S6 and Table S1).

To further evaluate the extent of polymerization control achieved for such PISA syntheses and to monitor the evolution in particle size and polydispersity, kinetic studies were conducted during the synthesis of GO_34_-H_1000_ diblock copolymer spheres at 37 °C when targeting 10% w/w solids. More specifically, aliquots were periodically withdrawn from the polymerizing mixture (every 30 min for the first 6 h and again after 8 h) and immediately quenched upon exposure to air, while cooling to 20 °C. These samples were then analyzed by ^1^H NMR spectroscopy in CD_3_OD for HPMA conversion calculations, SEC for determination of *M*_n_ and *M*_w_/*M*_n_, DLS for measurement of *D*_h_ and PD, as well as TEM for assessing the intermediate nanoparticle morphologies.

**Figure 3:**
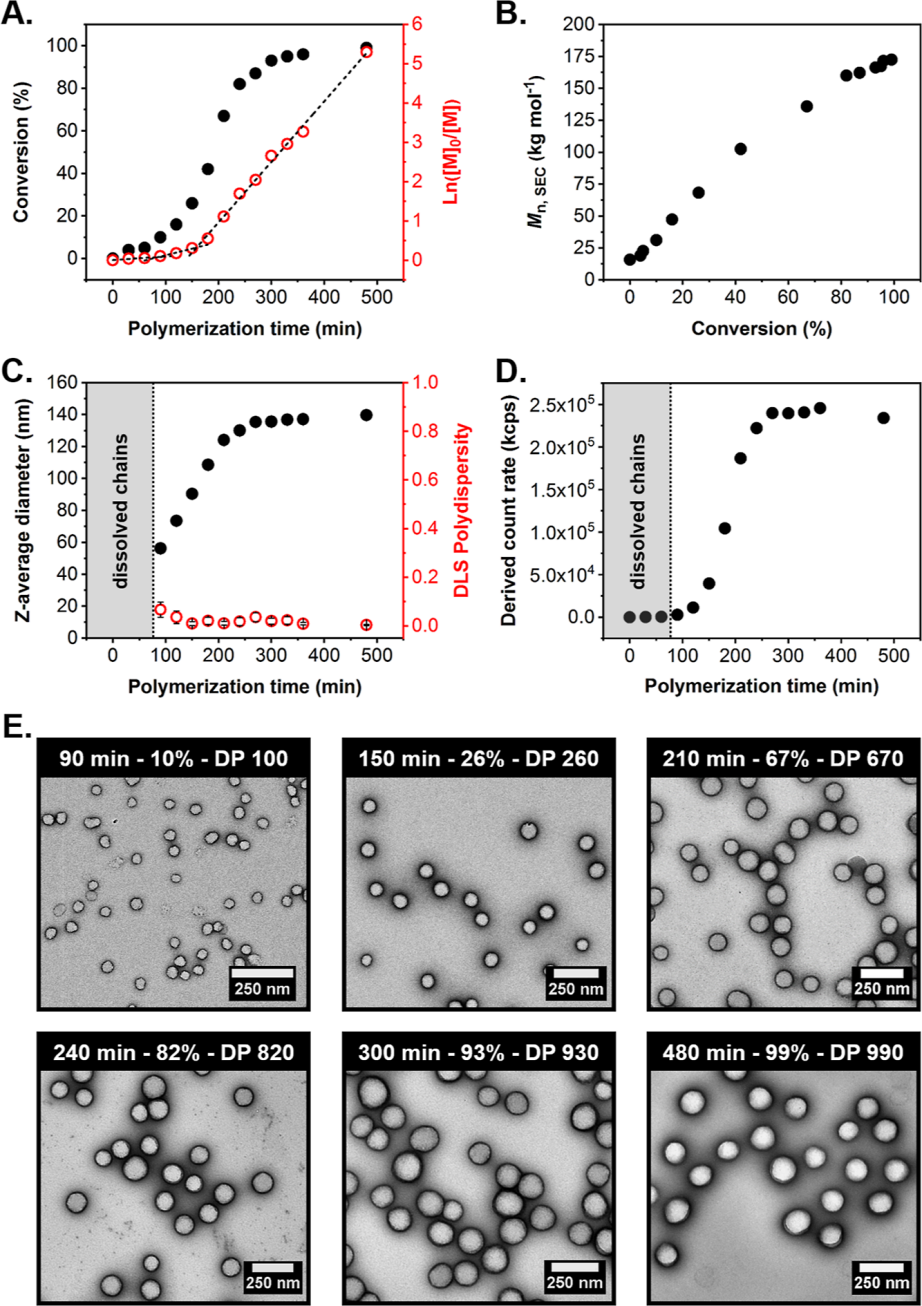
(A) HPMA conversion vs time curve (black filled circles) and corresponding ln([M]_0_/[M]) vs time plot (red empty circles), determined by ^1^H NMR spectroscopy during the synthesis of GO_34_-H_1000_ diblock copolymer spheres via RAFT aqueous dispersion polymerization of HPMA at 37 °C when targeting 10% w/w solids. (B) Evolution of *M*_n_ with increasing HPMA conversion for a series of intermediate GO_34_-H_*y*_ diblock copolymers prepared at 10% w/w solids, as calculated from SEC analysis (refractive index detector) using a series of PMMA calibration standards (DMF + 10 mM LiBr eluent). (C) Evolution of *z*-average diameter (black filled circles) and corresponding DLS polydispersity (PD) (red empty circles) during the polymerization, and (D) derived count rate vs time plot (black filled circles) for the same aqueous PISA formulation. (E) Representative TEM images recorded for GO_34_-H_*y*_ diblock copolymer nanoparticles formed at intermediate HPMA conversions, obtained using a uranyl formate stain. The polymerization time, instantaneous HPMA conversion, and corresponding PHPMA DP are indicated in each case.

On inspection of the constructed conversion vs time curve, almost all of the HPMA monomer (99%) had reacted within 8 h. The corresponding semi-logarithmic plot revealed a pseudo-first-order kinetic profile for this aqueous dispersion polymerization that was separated in three distinct reaction regimes (Figure 3A and Table S2). During the first 90 min, relatively slow RAFT solution polymerization occurred to produce water-soluble GO_34_-H_*y*_ diblock copolymer chains. After approximately 90 min—which corresponds to 10% monomer conversion, or a PHPMA DP of 100—a ∼3.5-fold increase in the rate of HPMA polymerization was observed, which indicated the onset of micellar nucleation. At this stage, the formed nascent micelles immediately become swollen with unreacted monomer, which leads to a relatively high local concentration and hence a much faster rate of polymerization.^45^ Finally, an additional four-fold rate enhancement was observed after ∼180 min (42% HPMA conversion, PHPMA DP = 420). This phenomenon has been reported for similar PISA formulations and is tentatively attributed to the formation of more compact nanoparticles via gradual expulsion of residual solvent molecules from the nanoparticle cores.^47,48^ Importantly, SEC analysis revealed a linear evolution in *M*_n_ with increasing monomer conversion, suggesting a controlled RAFT polymerization at each stage (Figure 3B and Table S2).

Further insights into the onset of micellar nucleation and size evolution were provided by DLS analysis of the same samples withdrawn at intermediate HPMA conversions from the reaction mixture. No particles were detected within the first 90 min, as judged by the relatively low scattering intensity (derived count rate <370 kcps), suggesting the initial formation of molecularly-dissolved copolymer chains (Figure 3C,D and Table S2). Once micellar nucleation occurred at 90 min (10% HPMA conversion), a linear increase in *D*_h_ was observed from 56 nm up to 124 nm over the following 120 min (67% HPMA conversion after 210 min), with a corresponding sequential increase in the derived count rate up to 186,700 kcps. As the remaining monomer within the nanoparticle cores is consumed, it is no longer replenished from the continuous phase. Additionally, as the growing nanoparticles become less swollen with the monomer, they also become denser (as low-density monomer is converted to higher-density polymer). Thus, only a rather modest increase in *D*_h_ and derived count rate was observed between this time point and the end of the polymerization. Notably, the *D*_h_ of 140 nm measured for the final GO_34_-H_1000_ diblock copolymer spheres produced during this kinetic study is comparable to that obtained for the respective sample with the same composition synthesized in the absence of any periodic sampling of the reaction mixture (*D*_h_ = 128 nm). However, the *D*_h_ values measured for the series of GO_34_-H_*y*_ diblock copolymer spheres isolated during this kinetic study are significantly higher than those observed when targeting compositionally similar diblock copolymers (e.g., *D*_h_ = 90 nm for GO_34_-H_260_ at 26% HPMA conversion vs *D*_h_ = 44 nm for GO_34_-H_250_ at >99% HPMA conversion) (Tables S1 and S2). This discrepancy is attributed to the monomer-swollen nature of the spheres in the former case. Importantly, DLS PDs remained below 0.07 for all kinetic samples, while TEM analysis confirmed the formation of near-monodisperse spheres of increasing size at higher HPMA conversions (Figures 3E and S7).

Recently, we reported that the pendent *cis*-diol functionality on PGEO5MA homopolymers, PGEO5MA-based diblock copolymer nano-objects, and PGEO5MA brushes can be selectively oxidized using sodium periodate (NaIO_4_) under mild reaction conditions to form the respective aldehyde-functional PAGEO5MA-based materials. The resulting PAGEO5MA chains can be further functionalized using various primary amines (including amino acids) via reductive amination.^32,33,35,36^ In the present study, our aim was to extend these reports by investigating whether dynamic imine bond formation could be used to promote the chemical adsorption of PAGEO5MA-stabilized diblock copolymer spheres of varying size onto surface-grafted amine-functionalized PDAGEO5MA brushes to produce a library of model soft hybrid surfaces of tuneable topography for friction coefficient measurements.

Accordingly, selective oxidation of the precursor GO_34_-H_*y*_ diblock copolymer spheres was achieved by addition of NaIO_4_ to 10% w/w aqueous copolymer dispersions at 22 °C in the dark for 20 min to yield a series of aldehyde-functional PAGEO5MA_34_-PHPMA_*y*_ (AGO_34_-H_*y*_) spheres, where *y* = 125-1500 (Scheme 1). A [*cis*-diol]/[NaIO_4_] molar ratio of unity was employed to ensure 100% oxidation of the PGEO5MA repeat units, as previously reported by Brotherton et al.^33^ Oxidized nanoparticles were subsequently diluted to 1% w/w solids prior to dialysis against deionized water to remove spent oxidant and the formaldehyde byproduct. ^1^H NMR spectroscopy analysis (CD_3_OD) confirmed the appearance of a new signal at approximately 3.5 ppm, whose intensity was proportional to the *cis*-diol content of each copolymer. This new feature is attributed to the formation of aldehyde groups in their hydrated germinal diol form, indicating the successful oxidation of the steric stabilizer chains (Figure S8).^32^ In principle, the secondary alcohol groups within the hydrophobic PHPMA block could be oxidized to form ketone groups. However, NaIO_4_ is highly selective toward *cis*-diol groups,^32,49^ so in practice this side reaction was not observed under the stated conditions.

Additionally, SEC analysis of the resulting AGO_34_-H_*y*_ diblock copolymers (DMF + 10 mM LiBr eluent) indicated a discernible increase in *M*_n_ and *M*_w_/*M*_n_ compared to the original GO_34_-H_*y*_ copolymers. Moreover, the development of high-molecular-weight shoulders led to significantly broader molecular-weight distributions, with such features being more pronounced for shorter PHPMA DPs (i.e., when the corona-forming PAGEO5MA block makes a larger contribution to the overall diblock copolymer molecular-weight distribution) (Figure S9 and Table S3). These observations are attributed to aldol cross-linking reactions occurring between the aldehyde and *cis*-diol groups of the stabilizer block chains at intermediate conversions during periodate oxidation or between the pendent aldehyde groups of PAGEO5MA and the hydroxyl groups of the partially hydrated PHPMA block.^33^

**Figure 4:**
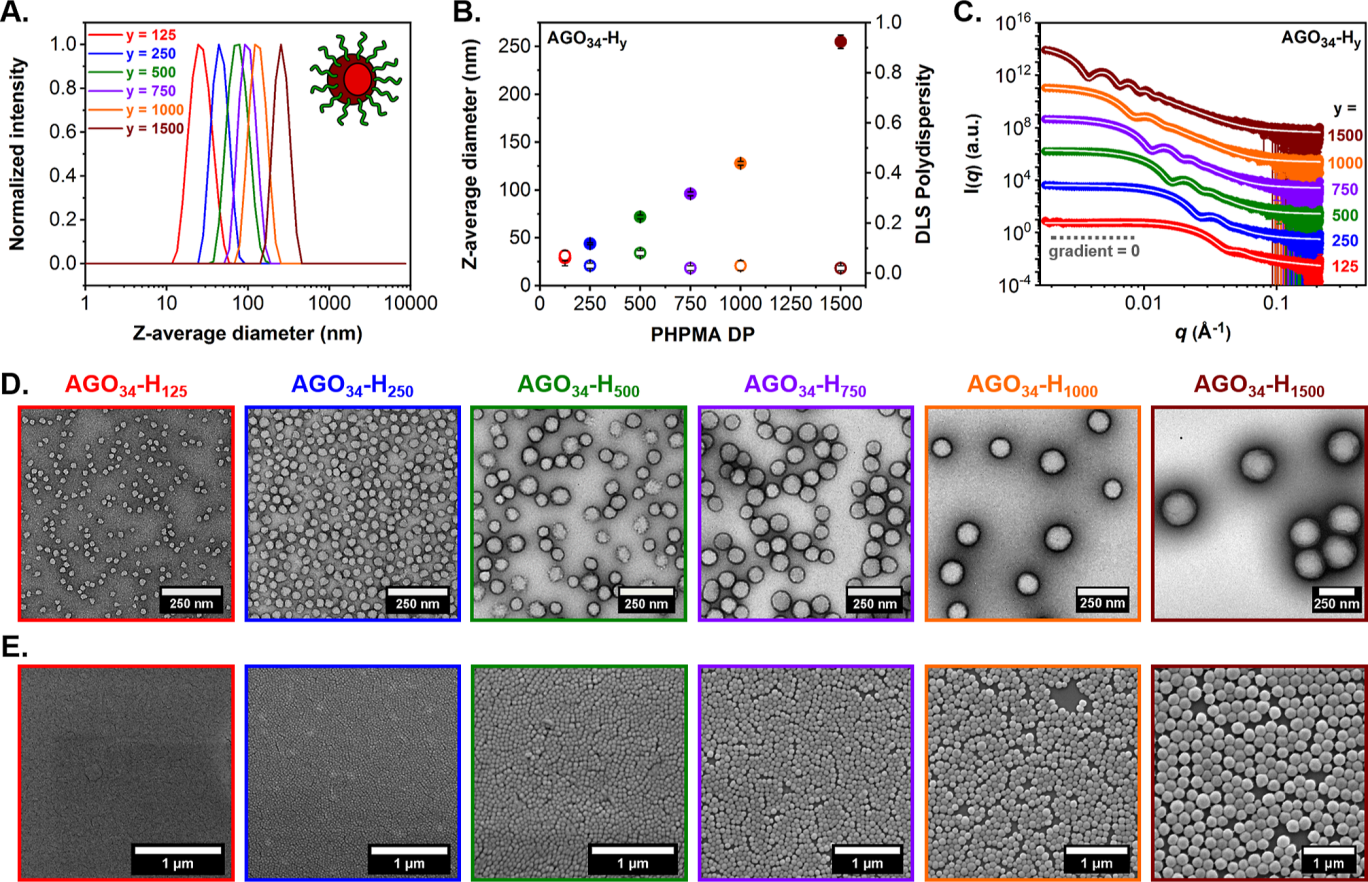
Summary of characterization data obtained for a series of aldehyde-functional AGO_34_-H_*y*_ diblock copolymer spheres (where *y* = 125–1500): (A) intensity-weighted DLS particle size distributions, (B) evolution of *z*-average diameter (filled circles) and corresponding DLS polydispersity (PD) (empty circles) with increasing target PHPMA DP, (C) SAXS patterns recorded for 1.0% w/w aqueous copolymer dispersions and corresponding data fits (white solid lines) applied to each SAXS pattern using a well-known spherical micelle model,^46^ (D) representative TEM images obtained using a uranyl formate stain, and (E) representative SEM images.

Importantly, DLS and SAXS analyses confirmed that periodate oxidation did not affect the particle size or colloidal stability of the developed AGO_34_-H_*y*_ diblock copolymer spheres. These nanoparticles exhibit almost identical *D*_h_ and *D*_SAXS_ diameters to those determined for the precursor GO_34_-H_*y*_ spheres prior to oxidation (Figures 4A–C and S11 and Table S3). These data were further supported by TEM, SEM, and AFM images, which indicated that near-monodisperse spherical nanoparticles of tuneable size were obtained for the whole diblock copolymer series (Figures 4D,E and S10–S12 and Table S3). Furthermore, no significant changes in zeta potential were observed by aqueous electrophoresis studies for the periodate-treated spheres (values ranged between −15 and −19.5 mV) (Figure S13 and Table S3).

**Scheme 2:**
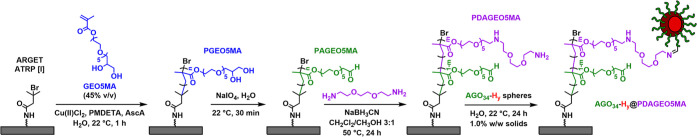
Schematic Illustration of the Grafting of PGEO5MA Homopolymer Brushes from Planar Silicon Wafers via SI-ARGET ATRP, Their Selective Oxidation to Produce Aldehyde-Functional PAGEO5MA Brushes, Their Subsequent Functionalization with a Hydrophilic Diamine (Jeffamine EDR-148) to Generate PDAGEO5MA Brushes, Followed by Chemical Adsorption of a Series of AGO_34_-H_*y*_ Diblock Copolymer Spheres (where *y* = 125–1500) onto Such PDAGEO5MA Brushes to Produce a Library of Nanoparticle-Decorated AGO_34_-H_*y*_@PDAGEO5MA Surfaces via DCC

So far we have demonstrated the synthesis of near-monodisperse kinetically-trapped AGO_34_-H_*y*_ spheres of increasing size. As highlighted above, our aim was to generate suitably modified PDAGEO5MA brushes to enable the chemical adsorption of such nanoparticles via dynamic imine bond formation. The synthetic route employed for the preparation of nanoparticle-decorated AGO_34_-H_*y*_@PDAGEO5MA hybrid surfaces is illustrated in Scheme 2.

Initially, ATRP initiator groups were introduced onto planar silicon wafers using a previously reported experimental protocol.^50^ Surface-initiated activators regenerated by electron transfer atom transfer radical polymerization (SI-ARGET ATRP) was then used to graft uniform PGEO5MA brushes of ∼120 nm dry thickness, as determined via ellipsometry (Table S4), from these modified substrates, following a procedure recently reported by our group.^36^ While, ultimately, aldehyde functionality is required for diamine functionalization and subsequent nanoparticle adsorption, *cis*-diol-functional PGEO5MA brushes were first grown herein as they allow for preparation of more densely grafted surfaces compared to brushes synthesized directly from polymerization of the respective AGEO5MA monomer.^36^

To produce the analogous aldehyde-functional PAGEO5MA brushes, such PGEO5MA brushes were exposed to an aqueous NaIO_4_ solution for 30 min at 22 °C (Scheme 2).^36^ Selective oxidation of the pendent *cis*-diol groups was confirmed by both ellipsometry and X-ray photoelectron spectroscopy (XPS) studies. Periodate oxidation should lead to a reduction in the molecular weight of each repeat unit owing to the loss of a formaldehyde molecule.^32^ Thus, given that the dry brush thickness is proportional to the polymer molecular weight,^51^ complete oxidation of the PGEO5MA repeat units is expected to produce an approximate 8.5% reduction in dry thickness.^36^ Indeed, ellipsometry measurements confirmed such a reduction in dry brush thickness equal to this theoretically expected value after exposing each PGEO5MA brush to NaIO_4_ (mean dry PAGEO5MA brush thickness = 111 nm) (Table S4). Moreover, comparing the C 1s XPS data obtained for the PGEO5MA and PAGEO5MA brushes revealed a shift in the ratio of the O–C=O, C=O, C–O–C/C–O–H, and C–H peaks that is consistent with brush oxidation (Figure S14).^36^

Recently, we have reported that PAGEO5MA-based chains,^32^ diblock copolymer nano-objects,^33,35^ and brushes^36^ can be further functionalized with various amino acids, such as l-histidine, via reductive amination. For surface-grafted PAGEO5MA brushes, this involves immersion into an aqueous solution containing the desired amine reagent and sodium cyanoborohydride (NaBH_3_CN), prior to heating to 50 °C for 24 h. Herein, this protocol was adapted to functionalize PAGEO5MA brushes using a hydrophilic diamine (Jeffamine EDR-148) to prepare the corresponding PDAGEO5MA brushes, as illustrated in Scheme 2. In principle, this diamine reagent could react with two aldehyde groups, leading to either intramolecular or intermolecular cross-linking. In practice, this potential issue was minimized herein by using a large excess of diamine.^52^ Furthermore, this derivatization reaction of the PAGEO5MA brushes with the diamine was performed in a binary solvent mixture (CH_2_Cl_2_/methanol 3:1), that is a poor solvent for the PAGEO5MA chains. Thus, the brushes were functionalized in their collapsed (rather than extended) conformation. This should limit diamine reaction with only the near-surface aldehyde groups of the brushes.^52−54^

**Figure 5:**
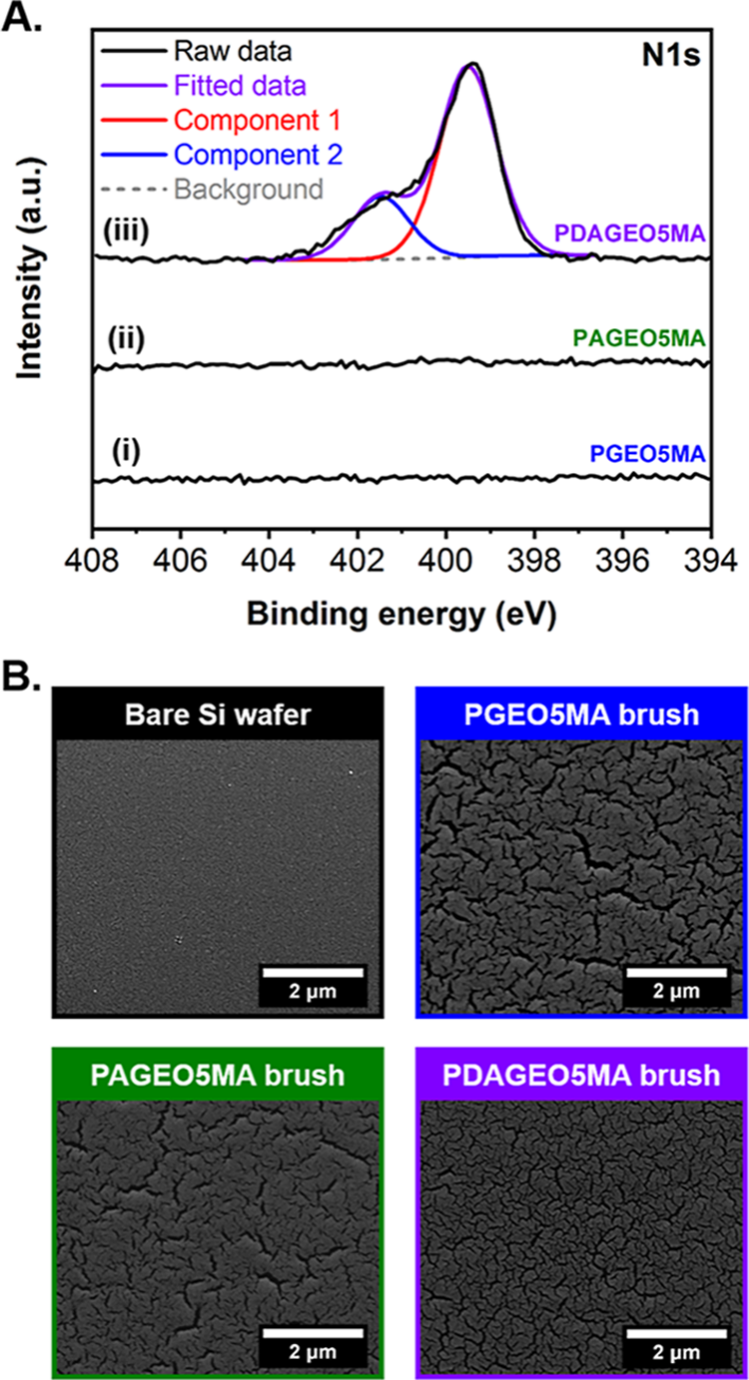
(A) High-resolution N 1s XPS data for surface-grafted (i) PGEO5MA (mean dry brush thickness = 121 nm), (ii) PAGEO5MA (mean dry brush thickness = 111 nm), and (iii) PDAGEO5MA (mean dry brush thickness = 129 nm) brushes (black lines). For the latter brush, the fitted data (purple line), individual components (red and blue lines), and subtracted background (gray dashed line) are also shown. (B) Representative SEM images recorded (after gold sputter coating) for a bare silicon wafer (black outline) and the surface-grafted PGEO5MA (blue outline), PAGEO5MA (green outline), and PDAGEO5MA (purple outline) dry brushes.

The formation of diamine-functional PDAGEO5MA brushes was verified by XPS (Figure 5A) and quantified by ellipsometry (Table S4). For the latter technique, the measured increase in dry brush thickness is compared to the maximum increase calculated for complete diamine conjugation (assuming no cross-linking side-reactions). More specifically, the molecular weight of the brush repeat units should increase from 351 g mol^–1^ for PAGEO5MA to 483 g mol^–1^ for PDAGEO5MA, which would result in a theoretical 38% increase in dry brush thickness. Hence, the mean degree of diamine functionalization throughout the entire PDAGEO5MA brush layer was calculated to be 36 ± 4% for the entire brush series (Table S4).^55^

XPS analysis was also employed to examine the surface composition of PDAGEO5MA brushes. The high-resolution N1s spectra for PGEO5MA, PAGEO5MA, and PDAGEO5MA brushes are shown in Figure 5A. As expected, the PGEO5MA and PAGEO5MA brushes exhibited no N 1s signal. However, a prominent N 1s signal was observed for the PDAGEO5MA brush, indicating successful diamine conjugation to the PAGEO5MA brush via reductive amination. The two distinct component peaks observed in the modeled XPS data correspond to N^+^ (blue line, 401.4 eV) and N^0^ (red line, 399.5 eV, indicating the presence of neutral and protonated amines.^54,56^ Each PDAGEO5MA brush was thoroughly rinsed with deionized water (pH 7) prior to drying for XPS analysis. The p*K*_a_ of the protonated primary amine of a similar Jeffamine molecule shifts from 9.7 in solution to 7.1 when grafted to a surface.^57^ A similar p*K*_a_ shift is observed for poly(tertiary amine methacrylate)-based brushes, whereby the p*K*_a_ of surface-grafted chains is significantly lower than that of either free chains or the corresponding monomer.^50,58−61^ Peak area analysis revealed a N^0^/N^+^ atomic ratio of approximately 3.0. Notably, this relatively low degree of amine protonation (25%) suggests a similar p*K*_a_ shift for the amine groups within the PDAGEO5MA brushes.

The degree of diamine functionalization was also examined by calculating the corresponding N/O atomic ratio. However, XPS only interrogates the upper ∼10 nm of the dry brush,^62^ so such data only provide the near-surface compositions, rather than throughout the whole brush layer. By comparing the experimental N/O atomic ratio to its maximum theoretical value (corresponding to 100% functionalization), the degree of functionalization was determined to be 56% for the upper surface of the PDAGEO5MA brush. This is significantly higher than the mean degree of functionalization calculated from ellipsometry measurements (36 ± 4%). This discrepancy provided evidence for the (partial) surface confinement of the diamine groups. This is not unexpected given that reductive amination was conducted in a poor solvent for the PAGEO5MA brush chains.^52−54^

SEM images acquired for a bare silicon wafer and representative examples of the surface-grafted PGEO5MA, PAGEO5MA, and PDAGEO5MA brushes are shown in Figure 5B. Clearly, a distinct change in the surface morphology was observed following the PGEO5MA brush grafting. The surface of each brush is relatively uniform, which is consistent with the corresponding ellipsometry data. However, multiple cracks were also observed for each polymer film. Since these features were not observed in the corresponding AFM images (Figure S15), they appear to be a drying artifact resulting either from the ultrahigh vacuum environment or the gold sputter coating required for SEM imaging.

Recently, we demonstrated the chemical adsorption of BSA onto a surface-grafted PAGEO5MA brush through the formation of multiple imine bonds between amines on the protein and the aldehyde groups on the polymer brush.^36^ Herein, we used a complimentary approach to adsorb a series of aldehyde-functional AGO_34_-H_*y*_ diblock copolymer spheres of varying size onto amine-functionalized PDAGEO5MA brushes via the same DCC.

**Figure 6:**
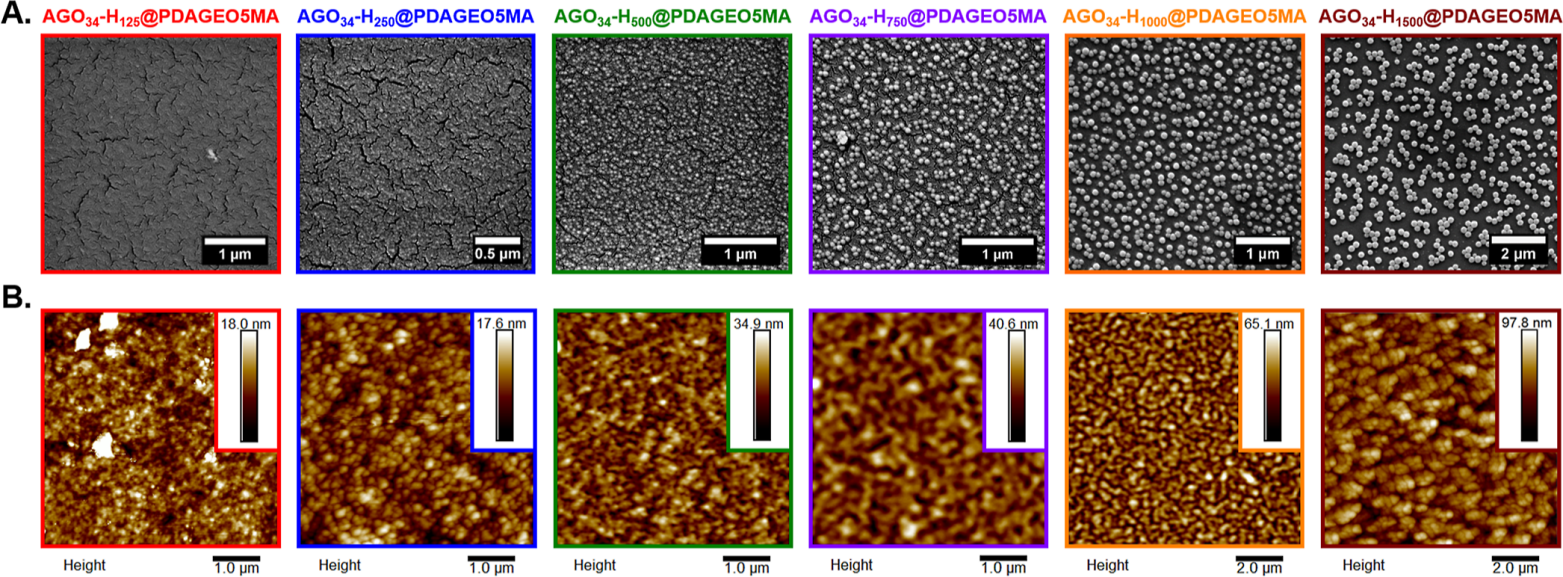
Representative (A) SEM and (B) AFM images (tapping mode) recorded for a series of nanoparticle-decorated AGO_34_-H_*y*_@PDAGEO5MA dry brushes (where *y* = 125, red outlines; *y* = 250, blue outlines; *y* = 500, green outlines; *y* = 750, purple outlines; *y* = 1000, orange outlines; and *y* = 1500, burgundy outlines). The mean AGO_34_-H_*y*_ nanoparticle hydrodynamic diameter (*D*_h_), as determined by DLS analysis, was 29 nm for *y* = 125, 44 nm for *y* = 250, 72 nm for *y* = 500, 96 nm for *y* = 750, 128 nm for *y* = 100, and 255 nm for *y* = 1500. Mean dry brush thickness for the initial PDAGEO5MA-based surfaces was 129 nm.

Nanoparticle adsorption onto PDAGE5OMA brushes was achieved via overnight immersion of the modified silicon wafers into 1% w/w aqueous diblock copolymer dispersions at 22 °C (Scheme 2). Representative SEM and AFM images of a series of the resulting AGO_34_-H_*y*_@PDAGEO5MA hybrid surfaces are shown in Figure 6A,B, respectively. Clearly, this facile dip-coating protocol led to efficient nanoparticle adsorption to the PDAGEO5MA brushes. The four largest AGO_34_-H_*y*_ diblock copolymer nanoparticle formulations (*y* = 500, 750, 1000, and 1500) could be distinctly observed via SEM, whereas the surface of the corresponding precursor PDAGEO5MA brush was essentially featureless (Figure 5B). Unfortunately, adsorption of the two smallest nanoparticle formulations (*y* = 125 and 250) could not be clearly visualized by SEM owing to insufficient resolution. However, AFM analysis confirmed the presence of all six types of nanoparticles at the brush surface (Figure 6B). Importantly, an AFM image of a bare dry PAGEO5MA brush indicated a relatively uniform topography with minimal surface roughness (Figure S15). In addition to visual confirmation of the adsorption of the AGO_34_-H_*y*_ diblock copolymer spheres, AFM height image analysis also revealed a significant increase in the roughness of each nanoparticle-decorated surface with increasing nanoparticle diameter.

The mean brush surface coverage by each nanoparticle sample was estimated by digital image analysis of the SEM images acquired for AGO_34_-H_*y*_@PDAGEO5MA surfaces coated with the four largest AGO_34_-H_*y*_ nanoparticles (*y* = 500–1500). This approach led to measurement of mean surface coverages of 31–38% (Figure S16). Such data are comparable to that reported in the literature for nanoparticle adsorption onto other polymer brushes^63−65^ or bare substrates^35,66,67^ via electrostatic interactions or covalent bond formation. Curiously, visual analysis of the AFM images acquired for the dried nanoparticle-coated surfaces indicated a notably higher degree of coverage. This discrepancy likely arises when further dehydration occurs on exposure of the samples to the ultrahigh vacuum during SEM imaging. This drying of the hybrid surfaces could result in particles separating on the surface, revealing the brush layer underneath. Given that XPS (Figures 5A and S14) indicated that 56% of the original aldehyde groups were converted into amines, such surface coverages suggest complete saturation of the available binding sites. Thus, despite the relatively weak nature of each individual imine bond (plus their propensity to undergo hydrolysis in aqueous solution), the DCC exploited herein leads to relatively efficient nanoparticle adsorption, presumably via formation of multiple imine bonds per nanoparticle. It is perhaps also worth mentioning that nanoparticle diffusion within a relatively dense brush layer is extremely unlikely, particularly for the larger nanoparticles.^68^ As such, DCC is almost certain surface-confined.

To confirm that dynamic Schiff base chemistry is responsible for the observed nanoparticle adsorption (rather than merely non-specific binding), two control experiments were conducted. First, a bare silicon wafer was exposed to AGO_34_-H_1000_ spheres (AGO_34_-H_1000_@Si). Second, a *cis*-diol-functional PGEO5MA brush was exposed to GO_34_-H_1000_ spheres (GO_34_-H_1000_@PGEO5MA) with SEM image analysis being used to quantify the extent of surface coverage in each case (Figure S17). Minimal nanoparticle adsorption onto the bare silicon wafer was observed (mean surface coverage ∼1%), whereas no detectable nanoparticle adsorption was observed for the PGEO5MA brush, hence excluding physical adsorption as a means of nanoparticle conjugation.

The anti-fouling capabilities of the *cis*-diol functional PGEO5MA brushes toward both proteins^36^ and sterically-stabilized nanoparticles (this work) highlight the importance of Schiff base chemistry in promoting nanoparticle adsorption. During their close approach to well-solvated polymer brush chains, such nanoparticles experience a repulsive force owing to the increase in osmotic pressure (as well as the concomitant reduction in entropic freedom).^69−72^ The formation of DCBs between the spheres and each polymer brush is clearly essential for particles to overcome this repulsive barrier.

Importantly, the topography of hydrated nanoparticle-decorated AGO_34_-H_y_@PDAGEO5MA surfaces was examined by AFM (Figure S18). Both the PDAGEO5MA brush chains and the steric stabilizer PAGEO5MA chains expressed at the surface of the adsorbed nanoparticles should become swollen when exposed to water. Nevertheless, in accordance with the analysis performed in dry conditions, the attachment of the nanoparticles was evident when compared to the bare PAGEO5MA hydrated brush (Figure S15). Moreover, there is a significant apparent increase in nanoparticle surface coverage. The continued presence of the nanoparticles on the brush surface following a drying-hydration cycle further highlighted DCC as the driving force for their adsorption.

Clearly, DCC can be exploited for the immobilization of polymer nanoparticles to surface-grafted polymer brushes to generate a library of composite interfaces. In principle, this approach could be extended to attach other polymer nano-objects, including worms and vesicles, opening these hybrid interfaces to a range of applications such as nanoreactor capture to surfaces and programmable surface lubrication. For each of these applications, an understanding of the surface topography and its interaction with other materials is required. In general, further development of this library of model nanoparticle-decorated brushes offers an opportunity to examine the role of topography on friction of soft surfaces, complementing existing alternative approaches.^73,74^ By investigating the correlation between surface topography and lateral force exerted on a probe, we can elucidate apparent disparities in surface coverage depicted in AFM and SEM images. Additionally, this will facilitate understanding of the interaction between nanoparticle-brush surfaces and extrinsic systems.

**Figure 7:**
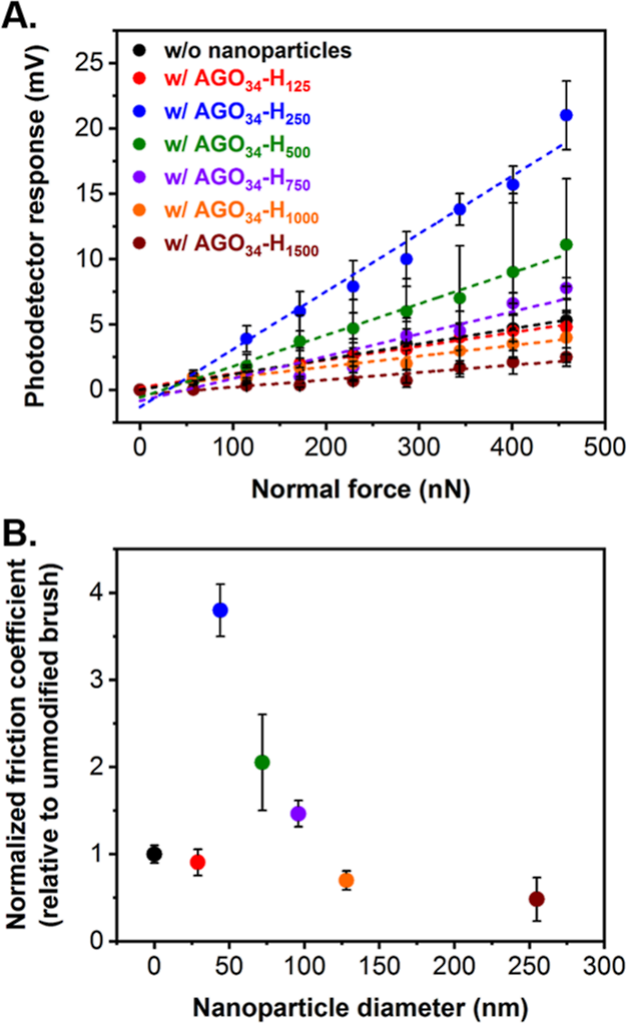
(A) AFM photodetector response as a function of applied normal force for a hydrated PAGEO5MA brush (black data) and a series of nanoparticle-decorated AGO_34_-H_*y*_@PDAGEO5MA hydrated surfaces (where *y* = 125, red data; *y* = 250, blue data; *y* = 500, green data; *y* = 750, purple data; *y* = 1000, orange data; and *y* = 1500, burgundy data), obtained by friction force microscopy analysis. In each case, the gradient obtained from the linear fits to each data set was used to determine the corresponding friction coefficient. (B) Normalized friction coefficients calculated for a series of nanoparticle-decorated AGO_34_-H_*y*_@PDAGEO5MA surfaces relative to that of a PAGEO5MA brush as a function of nanoparticle diameter.

The topography of each nanoparticle-brush hybrid surface was further examined by nanofriction measurements using friction force microscopy. The presence of the nanoparticles in the polymer film influences the lateral deflection of the AFM cantilever by modifying both the topography and the mechanical properties of the surface. Differences in specific interactions and adhesion are considered negligible since the steric stabilizer chains on the surface of the nanoparticles are chemically identical. Friction force microscopy studies were undertaken using the same AFM instrument. More specifically, each AGO_34_-H_*y*_@PDAGEO5MA brush was examined in aqueous solution at 24 °C. A single AFM tip (nominal radius of 10 nm) was used for all experiments, and the friction of each surface was normalized relative to a nanoparticle-free PAGEO5MA brush. Figure 7A shows the lateral photodetector response—which is proportional to the friction force—recorded for the AFM tip when increasing the normal load applied to each brush surface.

Friction force microscopy studies have been previously performed on polymer brushes.^75−77^ According to these prior studies, the total frictional force (*F*_F_) can be regarded as the sum of load-dependent (plowing) and area-dependent (shearing) terms.^78−80^ The balance of these two components determines the relationship between applied load and friction. In the present work, a linear response to increasing load was observed for all surfaces. Consequently, each data set was analyzed using Amontons’ law, *F*_F_ = μ*F*_N_, where *F*_N_ is the applied load and μ is the friction coefficient.^81^ Hence, the gradient of the data fit is equal to the friction coefficient. As a single tip was used for all surfaces, normalization of these coefficients to the nanoparticle-free PAGEO5MA brush (black data in Figure 7A) was straightforward. These relative values are shown in Figure 7B as a function of the nanoparticle diameter (measured by DLS). Measurement of such relative friction coefficients is both common and highly reproducible.^82,83^

For the smallest AGO_34_-H_125_ nanoparticles, the friction coefficient (μ) did not deviate significantly from that of the bare brush. However, a four-fold increase in this parameter was observed for the AGO_34_-H_250_@PDAGEO5MA brush. In this case, the nanoparticle radius (22 nm) is just over twice that of the nominal AFM tip radius (10 nm). However, as the nanoparticle radius increased further, a gradual reduction in the friction coefficient was observed with the AGO_34_-H_1000/1500_@PDAGEO5MA brushes exhibiting ∼50% of the bare PAGEO5MA brush friction coefficient.

**Scheme 3:**
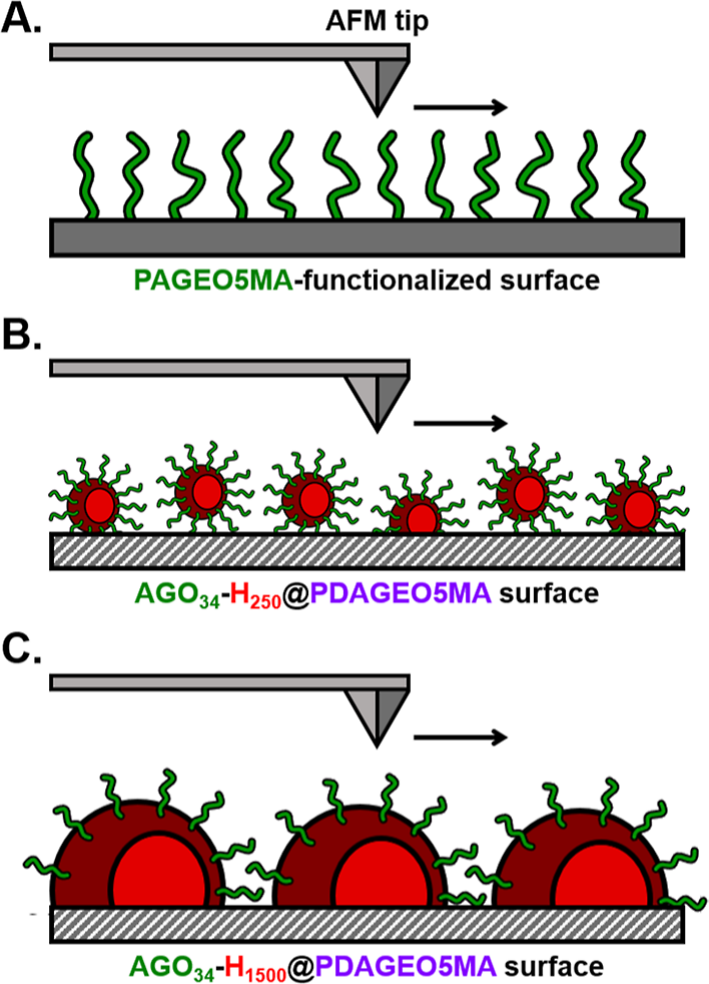
Schematic Illustration of the Interaction of the AFM Tip with (A) a Bare PAGEO5MA-Functionalized Surface, (B) an AGO_34_-H_250_@PDAGEO5MA Surface Decorated with Nanoparticles of Comparable Size to the AFM Tip, and (C) an AGO_34_-H_1500_@PDAGEO5MA Surface Decorated with Nanoparticles Significantly Larger in Size than the AFM Tip. According to Figure 7B, a Significant Increase in Relative friction Coefficient is Observed for Scenario B

These results are schematically presented in Scheme 3. For the nanoparticle-free PAGEO5MA-functionalized surface (Scheme 3A), the AFM probe tip simply shears over the surface, leading to a relatively low friction coefficient. For particles that are slightly larger than the AFM probe, a significant increase in the tip-sample contact area occurs at contact points between particles, leading to an increase in the rate of energy dissipation and hence in the friction force. However, as the particle size increases, the significance of these points of increased contact area decreases rapidly, and the friction coefficient reduces to a value slightly below that of the brush alone. The observed increase and subsequent decrease in the friction felt by the probe are thus ascribed to variations in contact surface area of the tip. Penetration of the tip into the interstices of particles increases the contact surface area, leading to additional adhesion and drag (Scheme 3B).^84^ For significantly larger nanoparticles, their diameter is much larger than the tip dimensions, and their interfacial curvature approaches that of the planar surface (Scheme 3C). The reduction in relative friction for brushes decorated with the largest nanoparticles is tentatively attributed to differences in elastic moduli between the steric stabilizer chains and the brush chains. This is not surprising given their differences in free volume.^85−87^ Given the complexity of these composite interfaces, a more nuanced modeling approach might reveal further details about the surfaces. In practice, Amonton’s law is sufficient to account for the observed change in photodetector response. As such, we adopted Occam’s razor and applied the simplest model to interpret the collected friction data.

Notably, surface forces apparatus is commonly employed to assess macroscopic surface friction.^88^ For example, vulcanized rubber coated with poly(methyl methacrylate) particles (at similar surface coverages to those presented herein) exhibited a monotonic reduction in friction with increasing particle size.^88^ Similarly, a significantly lower friction coefficient was observed within the boundary lubrication regime when conducting tribology experiments on epoxy-functionalized diblock copolymer nanoparticles covalently attached onto a planar stainless-steel substrate relative to comparable measurements performed using similar nanoparticles bearing no epoxy groups.^67^ Nevertheless, we posit that friction force studies via AFM are ideally suited for probing subtle differences in surface roughness for the nanoparticle-decorated brush surfaces reported herein. In this context, an interesting finding was reported by Peña-Parás et al., who used wear measurements to assess friction for a series of particle lubricants.^89^ This team found that using particles of intermediate size led to significantly less wear compared to both larger and smaller particles. This was attributed to the former particles being comparable in size to the surface roughness of the planar substrate. Thus, adsorption of such particles within the surface interstices reduced the overall surface roughness.

## Conclusions

DCC has been exploited to promote chemical adsorption of a series of aldehyde-functional AGO_34_-H_*y*_ diblock copolymer spheres (*y* = 125–1500) of varying size onto amine-functionalized polymer brushes. First, a series of *cis*-diol-functional precursor GO_34_-H_*y*_ diblock copolymer nanoparticles were prepared via RAFT aqueous dispersion polymerization and characterized by DLS, SAXS, TEM, SEM, and AFM. Well-defined spheres of increasing size were obtained when targeting higher PHPMA DPs. Kinetic studies during the synthesis of GO_34_-H_1000_ diblock copolymer spheres revealed a linear increase in *M*_n_ with HPMA conversion and a monotonic increase in particle *D*_h_ with polymerization time. Subsequently, selective oxidation of GO_34_-H_*y*_ nanoparticles using sodium periodate produced the corresponding aldehyde-functional AGO_34_-H_*y*_ nanoparticles with no discernible changes in either particle size or colloidal stability. In parallel studies, PGEO5MA precursor brushes were grafted from planar silicon wafers using surface-initiated ARGET ATRP. Ellipsometry and XPS analyses were used to confirm NaIO_4_ oxidation to afford the corresponding PAGEO5MA brushes and their subsequent chemical modification using a hydrophilic diamine in excess to generate amine-functionalized PDAGEO5MA brushes. This derivatization was deliberately performed in a poor solvent for the PAGEO5MA brush to ensure a surface-confined reaction. A control experiment confirmed that the precursor PGEO5MA brush was strongly resistant to nanoparticle adsorption. In contrast, the amine-functionalized PDAGEO5MA brushes readily formed imine bonds with the aldehyde-functional AGO_34_-H_*y*_ spheres. Thus DCC can be used to promote chemical adsorption of such nanoparticles. SEM analysis of AGO_34_-H_*y*_@PDAGEO5MA surfaces indicated a mean surface coverage of 33 ± 4% for the whole nanoparticle series. Moreover, friction force microscopy studies of such nanoparticle-decorated surfaces enabled calculation of friction coefficients, which were compared to that of a bare PAGEO5MA brush. Since the chemical structure of the steric stabilizer chains in the nanoparticles is the same regardless of their size, any observed variation in the friction coefficient can be attributed to changes in surface topography associated with nanoparticle adsorption. Reasonably consistent friction data were observed for all surfaces, except when the sphere diameter was comparable to the width of the AFM probe tip. In this case, the tip can penetrate between adjacent nanoparticles, which leads to a significant increase in contact area between the tip and the brush-nanoparticle layer and hence a higher friction coefficient. In principle, such systems provide useful model substrates for conducting friction force studies of soft surfaces. Moreover, the chemical adsorption of aldehyde-functional nanoparticles onto well-defined brushes via DCC is expected to be a generic approach. In particular, we are currently exploring the adsorption of enzyme-loaded diblock copolymer vesicles onto such brushes to produce surface-immobilized nanoreactors for enzyme-mediated catalysis applications.

## Experimental Section

Materials, characterization techniques, and detailed synthesis protocols are provided in the Supporting Information.

## Supporting Information Available

Materials and characterization techniques, synthetic protocols, ^1^H NMR spectrum and SEC curves for the GO_34_ precursor, characterization data for GO_34_-H_*y*_ and AGO_34_-H_*y*_ diblock copolymer spheres, additional TEM images obtained during kinetic studies for the synthesis of GO_34_-H_1000_ diblock copolymer spheres, and characterization data for bare polymer brushes and for brushes modified with (A)GO_34_-H_*y*_ diblock copolymer spheres (PDF)
