# Supplementary material for: Adsorption of Aldehyde-Functional Diblock Copolymer Spheres onto Surface-Grafted Polymer Brushes via Dynamic Covalent Chemistry Enables Friction Modification
Source: Chem Mater. 2023 Jul 19;35(15):6109–22. doi: 10.1021/acs.chemmater.3c01227 (PMC10413866; doi:10.1021/acs.chemmater.3c01227)
Supplement: Supplementary file 1 — cm3c01227_si_001.pdf [file cm3c01227_si_001.pdf]

# Supporting Information for:

# Adsorption of Aldehyde-Functional Diblock Copolymer Spheres onto Surface-Grafted Polymer Brushes *via* Dynamic Covalent Chemistry Enables Friction Modification

*Edwin C. Johnson,*[*,∇,a] *Spyridon Varlas,*[*,∇,a] *Oleta Norvilaite,*[a] *Thomas J. Neal,*[a] *Emma E. Brotherton,*[a] *George Sanderson,*[b] *Graham J. Leggett*[a] *and Steven P. Armes*[*,a]

[a] Department of Chemistry, University of Sheffield, Dainton Building, Brook Hill, Sheffield, S3 7HF, UK

[b] GEO Specialty Chemicals, Hythe, Southampton, SO45 3ZG, UK

[∇] E.C.J. and S.V. contributed equally to this work.

*Corresponding Authors: e.c.johnson@sheffield.ac.uk (E.C.J.), s.varlas@sheffield.ac.uk (S.V.), s.p.armes@sheffield.ac.uk (S.P.A.)

## Contents

Experimental Section ........ S2

Materials ........ S2

Characterization Techniques ........ S2

Synthesis Protocols ........ S6

Supplementary Characterization Data for the $GO_{34}$ Precursor ........ S11

Supplementary Characterization Data for $GO_{34}$-$H_y$ Diblock Copolymer Spheres ........ S12

Kinetic Studies during the Synthesis of $GO_{34}$-$H_{1000}$ Diblock Copolymer Spheres *via* Aqueous PISA.. S15

Supplementary Characterization Data for $AGO_{34}$-$H_y$ Diblock Copolymer Spheres ........ S17

Supplementary Characterization Data for PGEO5MA, PAGEO5MA, PDAGEO5MA and (A)$GO_{34}$-$H_y$-Decorated PDAGEO5MA Brushes ........ S22

References ........ S25

## Experimental Section

## Materials

4,4′-Azobis(4-cyanovaleric acid) (≥98.0%, ACVA), benzylamine (≥99.5%, distilled, Bz-$NH_2$), sodium periodate (≥99.8%, $NaIO_4$), (3-aminopropyl)triethoxysilane (>99%, APTES), triethylamine (99%, $NEt_3$), 2-bromoisobutyryl bromide (>99%, BIBB), copper(II) chloride (99.999%, $CuCl_2$), ascorbic acid (>98%, AscA), sodium cyanoborohydride (95%, $NaBH_3CN$), dichloromethane (>99%, DCM), methanol-$d_4$ (≥99.8%, $CD_3OD$) and lithium bromide (≥99%, LiBr) were purchased from Sigma-Aldrich, UK and were used without further purification. 2-Hydroxypropyl methacrylate (HPMA; mixture of 75 mol% 2-hydroxypropyl methacrylate and 25 mol% 2-hydroxyisopropyl methacrylate) was kindly donated by GEO Specialty Chemicals, UK and was used without further purification. GEO5MA monomer was synthesized according to our recently reported experimental protocol.[1] Jeffamine® EDR-148 was purchased from Huntsman, UK and was used as received. 2,2'-Azobis[2-(2-imidazolin-2-yl)propane]dihydrochloride (97%, VA-044) was purchased from Wako Chemicals, Japan. *N,N*-Dimethylformamide (≥99.9%, DMF) was purchased from VWR Chemicals, UK and was used without further purification. All other solvents were purchased from Fisher Scientific, UK and were used as received unless otherwise stated herein. Tetrahydrofuran (THF) was purchased from Fisher Scientific, UK and was dried over 4 Å molecular sieves (Acros Organics, UK) prior to use. *N,N,N',N'',N''*-Pentamethyldiethylenetriamine (>99%, PMDETA) and SnakeSkin™ dialysis tubing (MWCO = 3.5 kDa) were also purchased from Fisher Scientific, UK. 4-Cyano-4-(2-phenylethanesulfanylthiocarbonyl)sulfanylpentanoic acid (PETTC) and 4-cyano-4-(2-phenylethanesulfanylthiocarbonyl)sulfanylpentanoic succinimide ester (SPETTC) were synthesized according to previously reported experimental protocols.[2, 3] Deionized water (pH 6.8) was obtained using an Elga Elgastat Oprion 3A water purification system. Native oxide-coated silicon wafers were purchased from Pi-KEM, UK. Copper/palladium grids were purchased from Agar Scientific, UK and were carbon-coated in house.

## Characterization Techniques

***NMR Spectroscopy.*** $^1$H NMR spectra ($CD_3OD$) were recorded at 400 MHz using a Bruker Ascend™ 400 spectrometer. Chemical proton shifts are reported as $\delta$ in parts per million (ppm) and are expressed relative

to tetramethylsilane (TMS) at $\delta$ = 0 ppm when using $CDCl_3$ or the residual solvent peak at $\delta$ = 3.32 ppm when using $CH_3OD$.

***Size Exclusion Chromatography.*** Size exclusion chromatography (SEC) analysis was performed using an Agilent 1260 Infinity GPC system equipped with an Agilent guard column (PLgel 5 μm) and two Agilent Mixed-C columns (PLgel 5 μm), a differential refractive index (RI) and a UV-visible detector set to $\lambda$ = 305 nm. The mobile phase was DMF (HPLC grade) containing 10 mM LiBr at 60 °C at a flow rate of 1.0 mL $min^{-1}$. Number-average molecular weights ($M_n$), weight-average molecular weights ($M_w$) and dispersities ($Đ = M_w/M_n$) were calculated using a series of near-monodisperse poly(methyl methacrylate) (PMMA) calibration standards.

***Dynamic Light Scattering.*** Dynamic light scattering (DLS) analysis was performed using a Malvern Zetasizer Nano ZS instrument equipped with a 4 mW He–Ne 633 nm laser and an avalanche photodiode detector. Back-scattered light was detected at an angle of 173° and measurements were conducted at a copolymer concentration of 0.1% w/w at 25 °C. Malvern Zetasizer software v7.11 was used to calculate hydrodynamic diameters ($D_h$) *via* the Stokes-Einstein equation, which assumes perfectly monodisperse, non-interacting spherical particles. Data were averaged over at least three consecutive runs with at least ten measurements being recorded for each run.

***Aqueous Electrophoresis.*** Zeta potentials were determined *via* aqueous microelectrophoresis using the same Malvern Zetasizer Nano ZS instrument at 25 °C. Measurements were conducted at a copolymer concentration of 0.1% w/w in 1 mM KCl at pH 6.8 and data were averaged over at least three consecutive runs with at least ten measurements being recorded for each run. In each case, the zeta potential was calculated from the corresponding electrophoretic mobility ($\mu_E$) by using Henry's correction for the Smoluchowski equation ($\mu_E = 4\pi\ \varepsilon_0\ \varepsilon_r\ \zeta\ (1+\kappa r)/6\pi\ \mu$).

***Small-Angle X-Ray Scattering.*** SAXS patterns were recorded at a synchrotron facility (beamline I22 at Diamond Light Source, Didcot, Oxfordshire, UK). A monochromatic X-ray beam ($\lambda$ = 1.24 Å), 2D Dectris Pilatus 2M pixel detector and a scattering vector range of 0.002 $Å^{-1} < q <$ 0.210 $Å^{-1}$ were used for these experiments, where $q = \frac{4\pi}{\lambda}\sin\theta$ and $\theta$ is one-half of the scattering angle. Aqueous copolymer dispersions at 1.0% w/w were analyzed *via* a flow-through capillary set-up, using a glass capillary of 2.0 mm diameter. X-ray scattering data were reduced (integrated and normalized) using Dawn software supplied by Diamond Light Source. The X-ray scattering intensity for water was used for absolute scale calibration of

the obtained scattering patterns. Irena SAS macros for Igor Pro were utilized for background subtraction, modelling and further SAXS analysis.[4] SAXS patterns were fitted using a well-known spherical micelle model[5], which provides the mean core diameter and associated standard deviation.

***Transmission Electron Microscopy.*** Dry-state transmission electron microscopy (TEM) images were recorded at an acceleration voltage of 100 kV using a Philips CM100 microscope equipped with a Gatan 1k CCD camera. One 8 μL droplet of a 0.1% w/w aqueous copolymer dispersion was deposited *via* micropipette onto a plasma-treated carbon-coated copper/palladium grid at 25 °C. After roughly 1 min, excess sample was blotted from each grid using filter paper and the grid was subsequently stained by depositing an 8 μL droplet of a 0.75% w/w aqueous solution of uranyl formate for 1 min prior to blotting and drying under vacuum. Mean diameters for $GO_{34}$-$H_y$ and $AGO_{34}$-$H_y$ diblock copolymer spheres were determined by analyzing at least 100 nanoparticles in each case using ImageJ software.

***Scanning Electron Microscopy.*** Scanning electron microscopy (SEM) images were acquired using an FEI Inspect F field emission scanning electron microscope operating at an acceleration voltage of 5-15 kV. All $GO_{34}$-$H_y$ and $AGO_{34}$-$H_y$ diblock copolymer nanoparticle samples were prepared by drying dilute aqueous dispersions (0.1% w/w) onto silicon wafer chips at 25 °C. Brush-coated silicon wafers were imaged directly upon their purification. The sample-loaded silicon wafers were mounted on aluminium stubs using adhesive carbon tabs. Silver paint was applied to two edges of the mounted silicon wafers before sputter coating with a gold overlayer to prevent charge build-up. Surface coverages (%) for the brushes decorated with (A)$GO_{34}$-$H_y$ nanoparticles were assessed using ImageJ software and were averaged across multiple images recorded for each sample.

***Atomic Force Microscopy.*** Atomic force microscopy (AFM) imaging was performed using a Bruker Nanoscope™ VIII Multimode Atomic Force Microscope equipped with a 'J' scanner. Silicon cantilevers (OTESTPA-R3, Bruker, UK) with a nominal spring constant of 26 $N \cdot m^{-1}$ and tip radius of 9 nm were used for tapping mode imaging. Imaging of dry (A)$GO_{34}$-$H_y$ diblock copolymer spheres, dry or hydrated PAGEO5MA-functionalized and nanoparticle-decorated $AGO_{34}$-$H_y$@PDAGEO5MA brush surfaces was performed at 24 °C. All hydrated samples were allowed to equilibrate in solution for 20 min prior to analysis.

***Friction Force Microscopy.*** Lateral force data were collected using the same Bruker Nanoscope™ VIII Multimode Atomic Force Microscope. A single cantilever (CONTV, Bruker, UK) with a nominal tip

radius of 10 nm was used for all measurements. The spring constant of the cantilever was measured by first calibrating the photodetector sensitivity by measuring a force curve on a stiff bare silicon wafer. The spring constant was determined to be 0.2 $N{\cdot}m^{-1}$ from the thermal spectra within the Nanoscope™ software. Friction measurements for a series of nanoparticle-decorated $AGO_{34}$-$H_y$@PDAGEO5MA surfaces were performed in deionized water at 24 °C. A single line was captured throughout by disabling the slow scan axis. Measurements were made over a range of normal forces by sequentially increasing the normal force from 0 to 450 nN, followed by decreasing load in normal force until contact is lost. The scan frequency (1 Hz), scan size (1 μm) and samples per line (512) were kept constant across all samples and positions. The raw friction voltage was calculated by taking the average value of the middle 15% of the trace line scans, which corresponds to the full sliding regime. The lateral signal was converted into a force by multiplying by the lateral sensitivity and the spring constant. Normalized friction coefficient constants were calculated by dividing the gradient of the load-force curves of nanoparticle-decorated $AGO_{34}$-$H_y$@PDAGEO5MA brush surfaces to that of a bare PAGEO5MA brush.

***Ellipsometry.*** Ellipsometry measurements on dry polymer brushes grafted from planar silicon wafers were performed using a J. A. Woollam M-2000 V ellipsometer at a fixed angle of incidence of 75° normal to the sample surface in air at 20 °C. A wavelength range of $\lambda$ = 370 to 1000 nm was used to obtain two ellipsometry parameters ($\Psi$ and $\Delta$). These parameters were fitted to a two-layer model comprising a native oxide layer and Cauchy layer (**Equation S1**).

$$n(\lambda) = A_n = \frac{B_n}{\lambda^2} + \frac{C_n}{\lambda^4} \qquad \textbf{(S1)}$$

Data analysis and modelling were performed using Woollam CompleteEase software, which fits the $\Psi$ and $\Delta$ values calculated using this two-layer model to the experimental data. Cauchy constants of $A_n$ = 1.459, $B_n$ = 0.006, and $C_n$ = 0 were used for such analyses.

***X-Ray Photoelectron Spectroscopy.*** X-ray photoelectron spectroscopic (XPS) analysis of dry polymer brushes grafted from planar silicon wafers was performed using a Kratos Axis Supra spectrometer. Step sizes of 0.50 and 0.10 eV were used to record survey and high-resolution C1s, O1s and N1s spectra, respectively. In each case, spectra were recorded from at least two separate areas for each brush and were analyzed using Casa XPS software. All binding energies were calibrated with respect to the C1s saturated hydrocarbon signal at 285.0 eV.

## Synthesis Protocols

### Synthesis of Bz-PETTC RAFT agent

A dried round-bottom flask equipped with a magnetic stirrer bar was charged with SPETTC (1.0 g, 2.29 mmol), which was dissolved in anhydrous chloroform ($CHCl_3$; 50 mL). In a separate round-bottom flask, distilled benzylamine (0.23 g, 2.18 mmol) was dissolved in anhydrous $CHCl_3$ (5 mL), then added in one portion to the solution of SPETTC. The reaction mixture was heated at 30 °C for 16 h, filtered and washed with saturated $NaHCO_3$ solution (3 × 100 mL) to remove residual *N*-hydroxysuccinimide, before being dried using $MgSO_4$ (**Scheme S1**). After solvent removal *via* rotary evaporation, the resulting yellow oil was purified to remove any residual SPETTC *via* column chromatography using silica gel as the stationary phase and a 95:5 v/v $CH_2Cl_2$/methanol mixture as the eluent, followed by drying in a vacuum oven overnight to isolate the final product (Bz-PETTC) as a viscous yellow oil (0.78 g, 80%). $^1$H NMR (400 MHz, $CDCl_3$): $\delta$ (ppm) 7.22–7.36 (m, 10H, ***Ph***$CH_2$), 5.95–6.01 (d, 1H, (C=O)N***H***$CH_2$), 4.44 (d, 2H, PhC$\boldsymbol{H_2}$NH), 3.57 (m, 2H, Ph$CH_2$C$\boldsymbol{H_2}$S), 2.98 (m, 2H, PhC$\boldsymbol{H_2}$$CH_2$S), 2.49-2.53 (m, 4H, C($CH_3$)(C≡N)C$\boldsymbol{H_2}$C$\boldsymbol{H_2}$(C=O)), 1.89 (s, 3H, C$\boldsymbol{H_3}$).$^{13}$C NMR (400 MHz, $CDCl_3$): $\delta$ (ppm) 216.8 (***C***=S), 170.0 (***C***=O), 139.1, 137.8, 128.7, 127.9, 126.9 (***Ph***$CH_2$), 119.1 (SC($CH_3$)(***C***≡N)$CH_2$), 46.9 (S***C***($CH_3$)(C≡N)$CH_2$), 43.9 (Ph***C***$H_2$NH), 38.0 (Ph***C***$H_2$$CH_2$S), 34.5 (Ph$CH_2$***C***$H_2$S), 34.1 (***C***$H_2$$CH_2$(C=O)NH), 31.8 ($CH_2$***C***$H_2$(C=O)NH), 25.2 (***C***$H_3$).

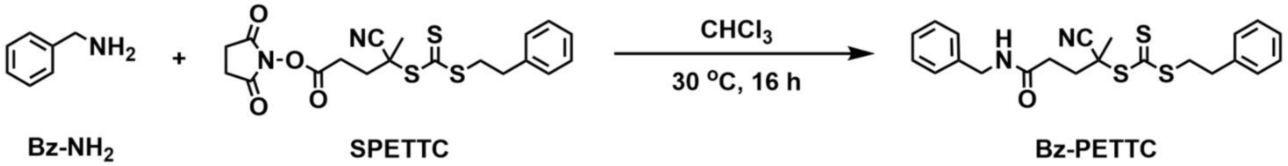


**Scheme S1.** Synthesis of Bz-PETTC by reaction of SPETTC with benzylamine in $CHCl_3$ at 30 °C.

### Synthesis of PGEO5MA$_{34}$ (or GO$_{34}$) precursor *via* RAFT solution polymerization of GEO5MA

The RAFT solution polymerization of GEO5MA was conducted according to previously reported experimental protocols.[1, 6] Briefly, GEO5MA (10.0 g, 26.3 mmol, 35 eq), Bz-PETTC (0.322 g, 0.75 mmol, 1.0 eq) and ACVA (0.042 g, 0.15 mmol, 0.20 eq; [Bz-PETTC]/[ACVA] molar ratio = 5.0) were dissolved in anhydrous ethanol (10.4 g, 50% w/w solids) in a round-bottom flask. The flask was then sealed and the resulting reaction mixture was purged with dry $N_2$(g) for 30 min prior to immersing the flask into a pre-heated oil bath at 70 °C for 180 min (**Scheme S2**). The polymerization was terminated by removing the

flask from the oil bath and cooling to 20 °C while exposing the reaction solution to air. A final GEO5MA conversion of 86% was determined by $^1H$ NMR analysis in $CD_3OD$. The resulting $GO_{34}$ precursor was isolated by precipitation into a ten-fold excess of diethyl ether, before being filtered and redissolved in methanol. This precipitation step was repeated, and the purified homopolymer was dried in a vacuum oven set at 35 °C overnight to produce a yellow viscous liquid. End-group analysis by $^1H$ NMR spectroscopy (400 MHz, $CD_3OD$) indicated a mean degree of polymerization (DP) of 34 for the purified PGEO5MA precursor. SEC analysis (DMF + 10 mM LiBr) using a refractive index detector and a series of PMMA calibration standards indicated an $M_n$ of 15.8 kg mol$^{-1}$ and an $M_w/M_n$ of 1.16.

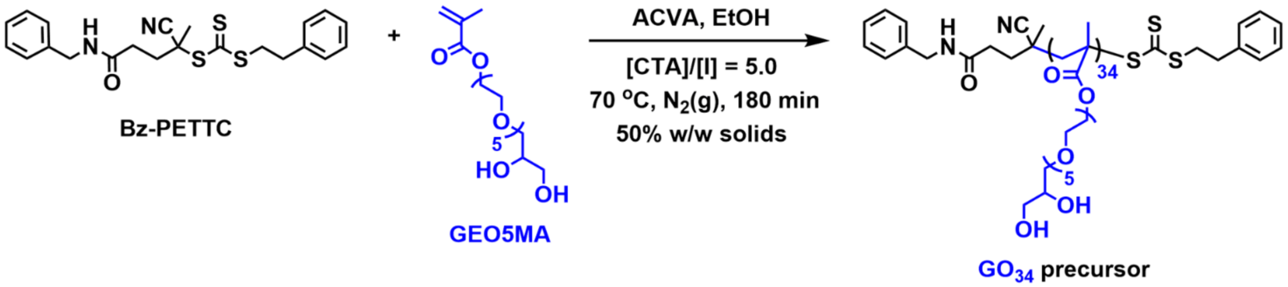


**Scheme S2.** Synthesis of a $GO_{34}$ precursor by RAFT solution polymerization of GEO5MA at 50% w/w solids in ethanol at 70 °C, using a Bz-PETTC RAFT agent.

**Synthesis of PGEO5MA$_{34}$-PHPMA$_y$ (or $GO_{34}$-$H_y$) diblock copolymer spheres *via* RAFT aqueous dispersion polymerization of HPMA**

A typical experimental procedure followed for the synthesis of $GO_{34}$-$H_{500}$ diblock copolymer spheres at 10% w/w solids by RAFT aqueous dispersion polymerization of HPMA was conducted as follows. HPMA (0.135 g, 0.94 mmol, 500 eq), $GO_{34}$ precursor (0.025 g, 1.87 µmol, 1.0 eq) and VA-044 initiator (0.2 mg, 0.62 µmol, 0.33 eq – 10 µL aliquot from a 10× concentrated solution; [$GO_{34}$ macro-CTA]/[VA-044] molar ratio = 3.0) were placed in a glass vial equipped with a magnetic stirrer bar and dispersed in deionized water (1.44 mL, 10% w/w solids). The vial was then sealed, purged with dry $N_2$(g) for 20 min and immersed in a pre-heated oil bath at 37 °C. After 16 h, the HPMA polymerization was terminated by removing the vial from the oil bath and cooling to 20 °C while exposing the reaction solution to air. The final HPMA conversion was >99%, as judged by $^1H$ NMR spectroscopy (400 MHz, $CD_3OD$). Similar protocols were also adopted for the synthesis of a series of $GO_{34}$-$H_y$ spheres (where y = 125, 250, 750, 1000 or 1500) at 10% w/w by adjusting the respective amounts of monomer, precursor, initiator and solvent required in each case.

**Kinetic studies during the synthesis of $GO_{34}$-$H_{1000}$ diblock copolymer spheres *via* RAFT aqueous dispersion polymerization**

The experimental protocol employed for kinetic studies during the aqueous PISA synthesis of $GO_{34}$-$H_{1000}$ spheres at 10% w/w solids is described. HPMA (0.863 g, 5.99 mmol, 1000 eq), $GO_{34}$ precursor (0.080 g, 5.99 μmol, 1.0 eq) and VA-044 initiator (0.6 mg, 1.98 μmol, 0.33 eq – 10 μL aliquot from a 10× concentrated solution; [$GO_{34}$ macro-CTA]/[VA-044] molar ratio = 3.0) were placed in a round-bottom flask equipped with a magnetic stirrer bar and dispersed in deionized water (8.49 mL, 10% w/w solids). The flask was then sealed, purged with dry $N_2$(g) for 20 min and immersed in a pre-heated oil bath at 37 °C for 16 h. Aliquots (~200 μL) were withdrawn from the polymerizing mixture every 30 min for the first 6 h and once more after 8 h. Each aliquot was quenched *via* immediate dilution using (a) $CD_3OD$ for $^1$H NMR spectroscopic analysis to calculate the HPMA conversion, (b) DMF (+ 10 mM LiBr) for SEC analysis, or (c) deionized water for DLS and TEM analysis. After 16 h, the HPMA polymerization was terminated by removing the flask from the oil bath and cooling to 20 °C, while exposing the reaction solution to air. The final aqueous copolymer dispersion was characterized by $^1$H NMR, SEC, DLS and TEM.

**Selective oxidation of *cis*-diol-functional PGEO5MA$_{34}$-PHPMA$_y$ (or $GO_{34}$-$H_y$) diblock copolymer spheres to produce aldehyde-functional PAGEO5MA$_{34}$-PHPMA$_y$ (or AGO$_{34}$-$H_y$) diblock copolymer spheres**

The selective oxidation of $GO_{34}$-$H_y$ spheres was conducted according to a previously reported experimental protocol.[6] A typical experimental protocol employed for the oxidation of PGEO5MA$_{34}$-PHPMA$_{500}$ diblock copolymer spheres using sodium periodate was conducted as follows. $NaIO_4$ (4.26 mg, 19.90 μmol) was dissolved in a 10% w/w aqueous dispersion of PGEO5MA$_{34}$-PHPMA$_{500}$ diblock copolymer spheres (0.50 mL, 50 mg (or 0.59 μmol) of copolymer, 19.90 μmol *cis*-diol functionality). A $NaIO_4$/*cis*-diol molar ratio of unity was used to target 100% oxidation of the PGEO5MA$_{34}$ block. The reaction solution was stirred in the dark for 20 min at 22 °C and was subsequently diluted with deionized water to 1% w/w solids content before transferring it into a dialysis bag (MWCO = 3.5 kDa) and dialyzing against deionized water for 24 h with periodic changes of the external medium to remove any unreacted $NaIO_4$. Similar protocols were also adopted for the oxidation of a series of $GO_{34}$-$H_y$ diblock copolymer spheres (where y = 125, 250, 750, 1000 or 1500) at 10% w/w by adjusting the mass of $NaIO_4$ required in each case.

**Surface functionalization of planar silicon wafers with ARGET ATRP initiator groups**

Functionalization of planar silicon wafers with ARGET ATRP initiator moieties was conducted using a modified experimental protocol previously reported by Johnson *et al.*[7] Silicon (100) wafers were cut into small pieces (~1 × 1 $cm^3$) before being UV-ozone cleaned for 30 min using a Bioforce Nanosciences ProCleaner. The treated wafers were placed in an open petri-dish along with a 3 mL glass vial containing ~100 µL APTES. The petri-dish was then placed in a desiccator, which was subsequently sealed and placed under vacuum. Vapor deposition was allowed to progress for 30 min before the wafers were removed and placed in an oven heated at 110 °C for 30 min. The wafers were then functionalized by immersion in $CH_2Cl_2$ followed by sequential addition of $NEt_3$ (final concentration = 0.2 M) and BIBB (final concentration = 0.2 M), allowing the surface amidation reaction to proceed for 1 h at 22 °C. Finally, the initiator-functionalized silicon wafers were rinsed extensively with ethanol and deionized water, before drying under a stream of compressed air.

**Synthesis of PGEO5MA brushes *via* surface-initiated ARGET ATRP**

Following surface functionalization with ATRP initiator groups, surface-initiated ARGET ATRP was employed to graft PGEO5MA brushes from each wafer.[8] Briefly, a GEO5MA:$CuCl_2$:PMDETA:AscA molar ratio of 1000:1:5:10 was used (21.6 g GEO5MA; 7.6 mg $CuCl_2$; 49.2 mg PMDETA; 0.1 g AscA). Deionized water (22.0 g) was added to afford a final monomer concentration of 45% v/v. Polymerizations were allowed to proceed for 1 h at 22 °C in all cases. Each PGEO5MA-functionalized silicon wafer was rinsed thoroughly with ethanol and deionized water and then dried using a stream of $N_2$ gas. The kinetics of such surface-initiated polymerizations differ from those for the analogous solution polymerizations[9, 10] and the determination of molecular weight for the PGEO5MA chains *via* brush degrafting is not feasible (owing to the small mass of recovered polymer). Thus, the brush grafting density was assumed to be comparable to earlier brushes prepared using similar synthesis protocols.[11-13]

**Selective oxidation of *cis*-diol-functional PGEO5MA brushes to produce aldehyde-functional PAGEO5MA brushes**

Following our previously reported experimental protocol for selective oxidation of surface-grafted PGEO5MA brushes,[8] PGEO5MA-functionalized planar silicon wafers were immersed in a 3.0 mg $mL^{-1}$ aqueous solution of $NaIO_4$ for 30 min at 22 °C, targeting full oxidation in all cases. Each PAGEO5MA-

functionalized silicon wafer was rinsed thoroughly with deionized water and then dried using a stream of compressed air.

**Preparation of diamine-functional PDAGEO5MA brushes from aldehyde-functional PAGEO5MA brushes *via* reductive amination**

PAGEO5MA brushes were functionalized in their collapsed state using a bifunctional diamine (Jeffamine® EDR-148) *via* reductive amination. A 3.0 mg $mL^{-1}$ solution of this diamine was prepared using a 3:1 $CH_2Cl_2$/methanol solvent mixture, which is a poor solvent for PAGEO5MA, prior to addition of 1.5× molar excess of $NaBH_3CN$. Brushes were immersed in this reaction solution for 24 h at 50 °C. The resulting PDAGEO5MA-functionalized wafers were rinsed extensively with methanol and deionized water and then dried under a stream of compressed air.

**Adsorption of (A)$GO_{34}$-$H_y$ diblock copolymer spheres onto surface-grafted polymer brushes**

In a typical experiment, PDAGEO5MA-functionalized wafers were immersed into 1% w/w aqueous dispersions of dialyzed $AGO_{34}$-$H_y$ diblock copolymer spheres (where y = 125, 250, 500, 750, 1000 or 1500). Adsorption *via* Schiff base chemistry (i.e., imine bond formation) was allowed to proceed for 24 h at 22 °C. Nanoparticle-decorated $AGO_{34}$-$H_y$@PDAGEO5MA surfaces were then rinsed thoroughly with deionized water and dried under a stream of compressed air. Similar protocols were adopted for control experiments to confirm the relatively weak physical adsorption of (i) $AGO_{34}$-$H_y$ spheres onto a bare silicon wafer and (ii) $GO_{34}$-$H_y$ spheres onto a bare PGEO5MA brush.

## Supplementary Characterization Data for the $GO_{34}$ Precursor

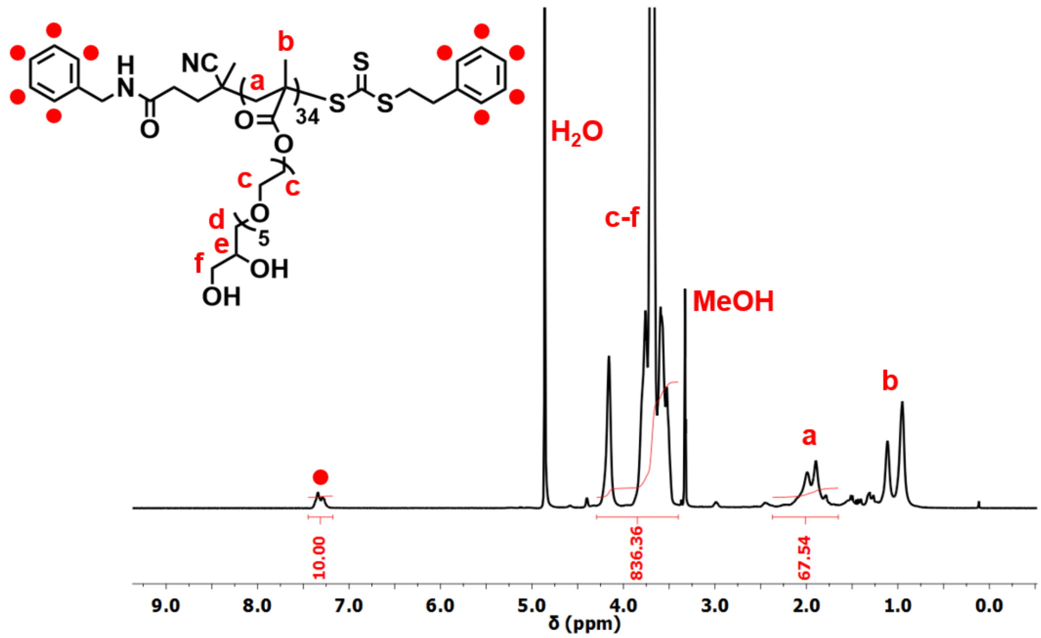


**Figure S1.** Assigned [1]H NMR spectrum ($CD_3OD$) recorded for the purified $GO_{34}$ precursor prepared *via* RAFT solution polymerization of GEO5MA using a Bz-PETTC RAFT agent.

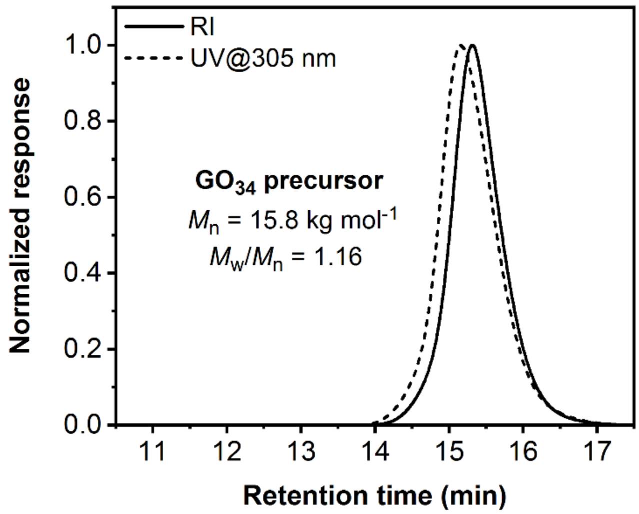


**Figure S2.** Normalized SEC curves recorded for the $GO_{34}$ precursor using RI (solid line) and UV (dashed line, $\lambda$ = 305 nm) detectors, along with the corresponding $M_n$ and $M_w/M_n$ values calculated using a series of PMMA calibration standards (DMF + 10 mM LiBr eluent).

## Supplementary Characterization Data for $GO_{34}$-$H_y$ Diblock Copolymer Spheres

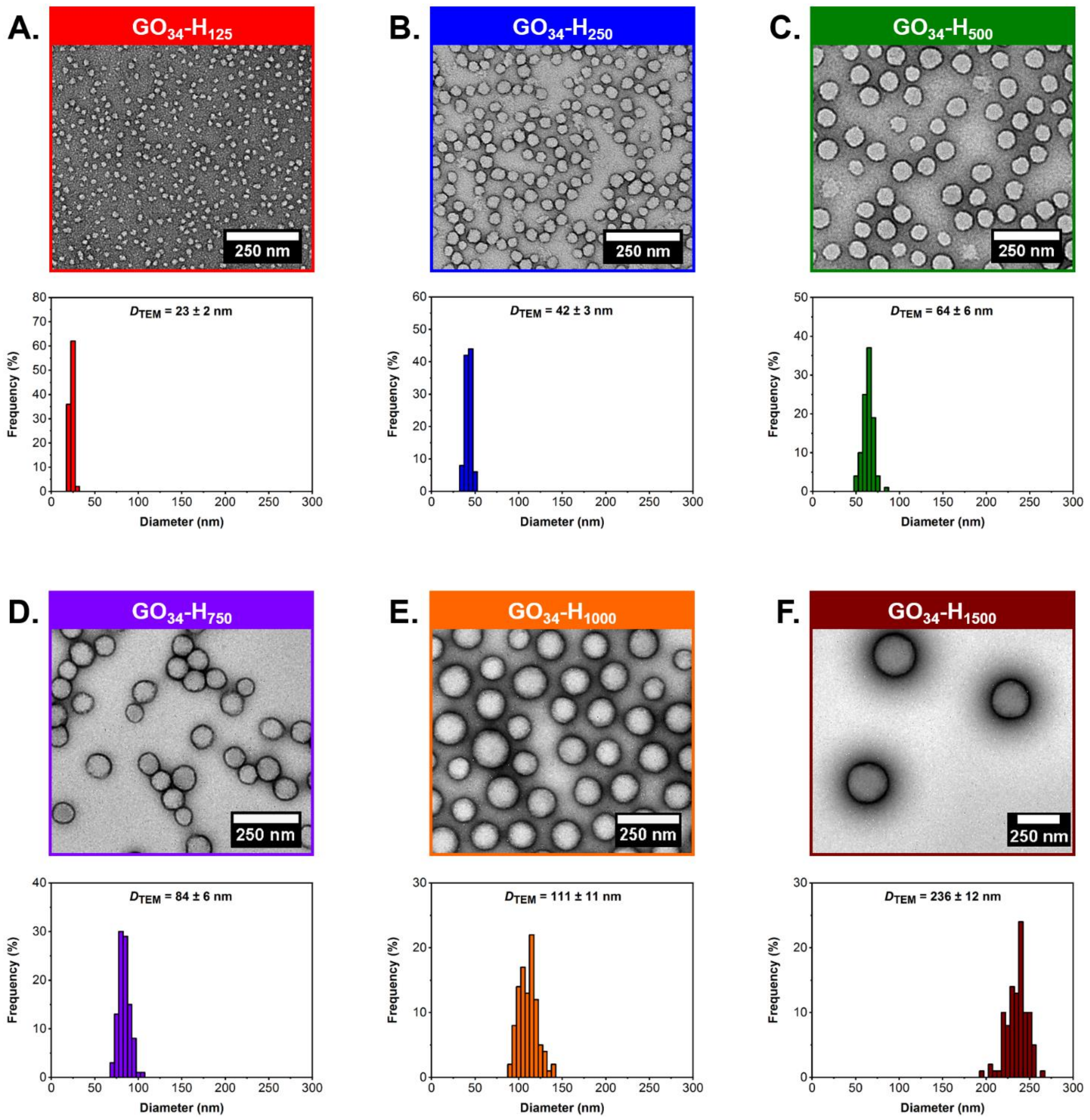


**Figure S3.** Representative TEM images recorded for $GO_{34}$-$H_y$ diblock copolymer spheres, where (A) y = 125; (B) y = 250; (C) y = 500; (D) y = 750; (E) y = 1000; and (F) y = 1500, using a 0.75% w/w uranyl formate stain, and corresponding size distribution histograms, with the mean diameter ($D_{TEM}$), determined by analyzing at least 100 nanoparticles in each case.

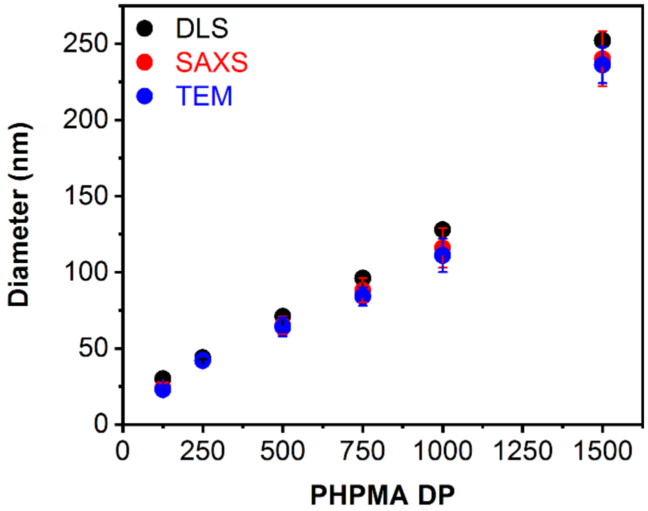


**Figure S4.** Comparison of mean diameters for a series of $GO_{34}$-$H_y$ diblock copolymer spheres determined by DLS (black circles), SAXS (red circles) and TEM analysis (blue circles).

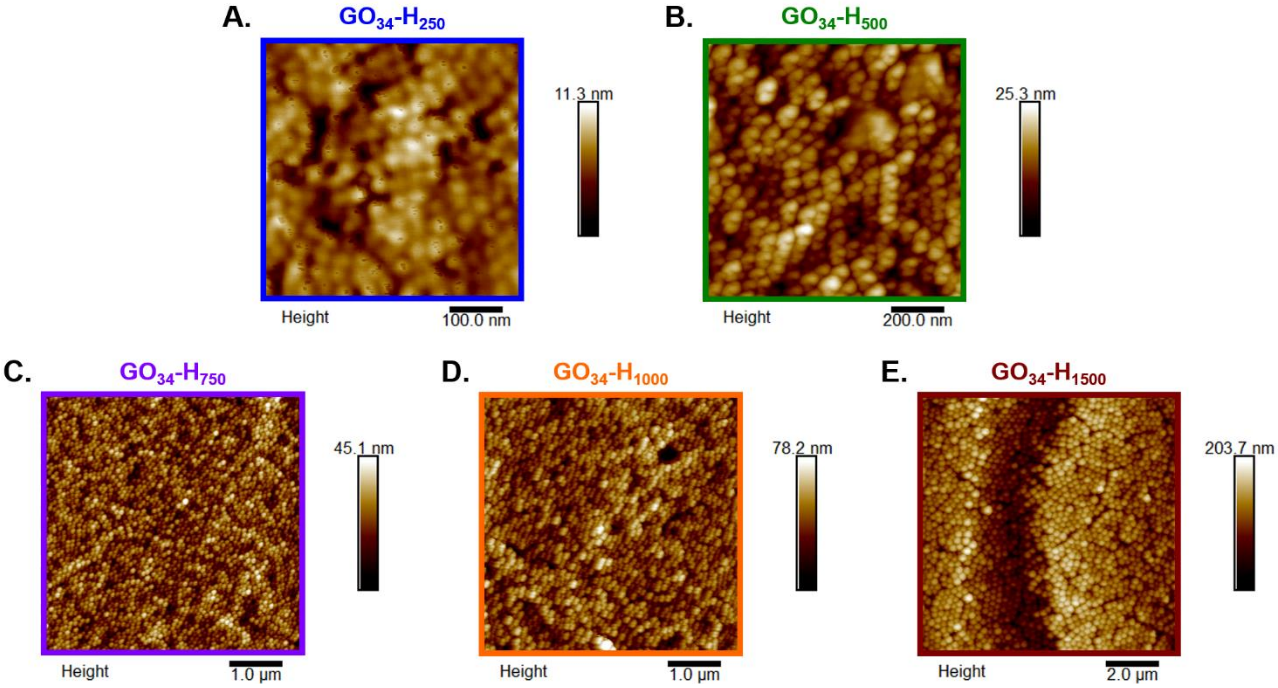


**Figure S5.** Representative AFM images recorded for a series of dried $GO_{34}$-$H_y$ diblock copolymer spheres prepared *via* aqueous PISA at 10% w/w solids, where (A) y = 250; (B) y = 500; (C) y = 750; (D) y = 1000; and (E) y = 1500.

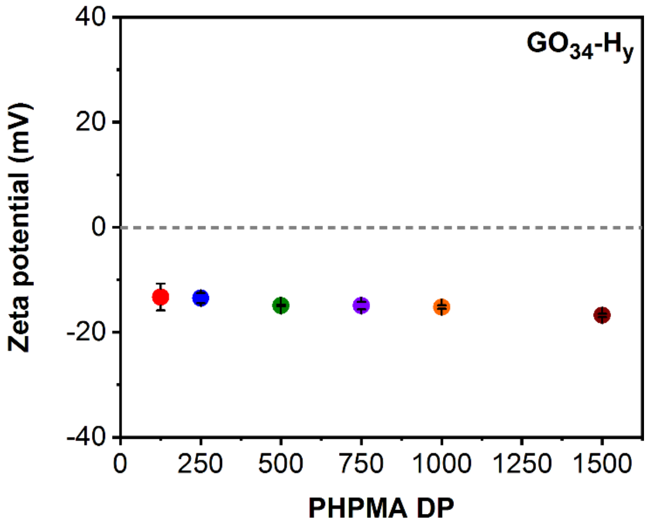


**Figure S6.** Variation in zeta potential with increasing target PHPMA DP for a series of $GO_{34}$-$H_y$ diblock copolymer spheres prepared *via* aqueous PISA at 10% w/w solids, as determined by aqueous electrophoresis analysis in 1 mM KCl at pH 6.8.

**Table S1.** Summary of monomer conversions, SEC data, DLS data, zeta potentials, SAXS and TEM mean diameters for a series of *cis*-diol-functional $GO_{34}$-$H_y$ diblock copolymer spheres prepared *via* RAFT aqueous dispersion polymerization of HPMA.

| Copolymer | [solids] (% w/w) | HPMA conversion[a] (%) | $M_{n, SEC}$[b] (kg mol$^{-1}$) | $M_w/M_n$[b] | $D_h$[d] (nm) | PD[d] | ζ-Potential[e] (mV) @ pH 6.8 | $D_{SAXS}$[f] (nm) | $D_{TEM}$[g] (nm) |
|---|---|---|---|---|---|---|---|---|---|
| **$GO_{34}$-$H_{125}$** | 10 | >99 | 36.3 | 1.18 | 30 ± 1 | 0.02 ± 0.01 | - 13.3 ± 2.5 | 24 ± 3 | 23 ± 2 |
| **$GO_{34}$-$H_{250}$** | 10 | >99 | 57.8 | 1.34 | 44 ± 1 | 0.01 ± 0.01 | - 13.5 ± 1.0 | 42 ± 3 | 42 ± 3 |
| **$GO_{34}$-$H_{500}$** | 10 | >99 | 94.8 | 1.61 | 71 ± 1 | 0.08 ± 0.02 | - 14.9 ± 0.2 | 65 ± 6 | 64 ± 6 |
| **$GO_{34}$-$H_{750}$** | 10 | >99 | 131.9 | 1.94 | 96 ± 1 | 0.01 ± 0.01 | - 14.9 ± 0.7 | 88 ± 8 | 84 ± 6 |
| **$GO_{34}$-$H_{1000}$** | 10 | >99 | 159.3 | 2.49 | 128 ± 2 | 0.03 ± 0.02 | - 15.2 ± 0.3 | 116 ± 13 | 111 ± 11 |
| **$GO_{34}$-$H_{1500}$** | 10 | >99 | -[c] | -[c] | 252 ± 4 | 0.04 ± 0.04 | - 16.8 ± 0.3 | 240 ± 18 | 236 ± 12 |

[a]Monomer conversion calculated from $^1$H NMR spectroscopy in $CD_3OD$. [b]$M_n$ and $M_w/M_n$ data calculated by SEC RI analysis relative to a series of PMMA calibration standards (DMF + 10 mM LiBr eluent). [c]Poor solubility in SEC eluent. [d]$D_h$ and polydispersities determined by DLS analysis. [e]Zeta potentials determined by aqueous electrophoresis conducted in 1 mM KCl at pH 6.8. [f]$D_{SAXS}$ determined by fitting the corresponding SAXS patterns. [g]$D_{TEM}$ determined from TEM images by analyzing at least 100 nanoparticles in each case.

## Kinetic Studies during the Synthesis of $GO_{34}$-$H_{1000}$ Diblock Copolymer Spheres *via* Aqueous PISA

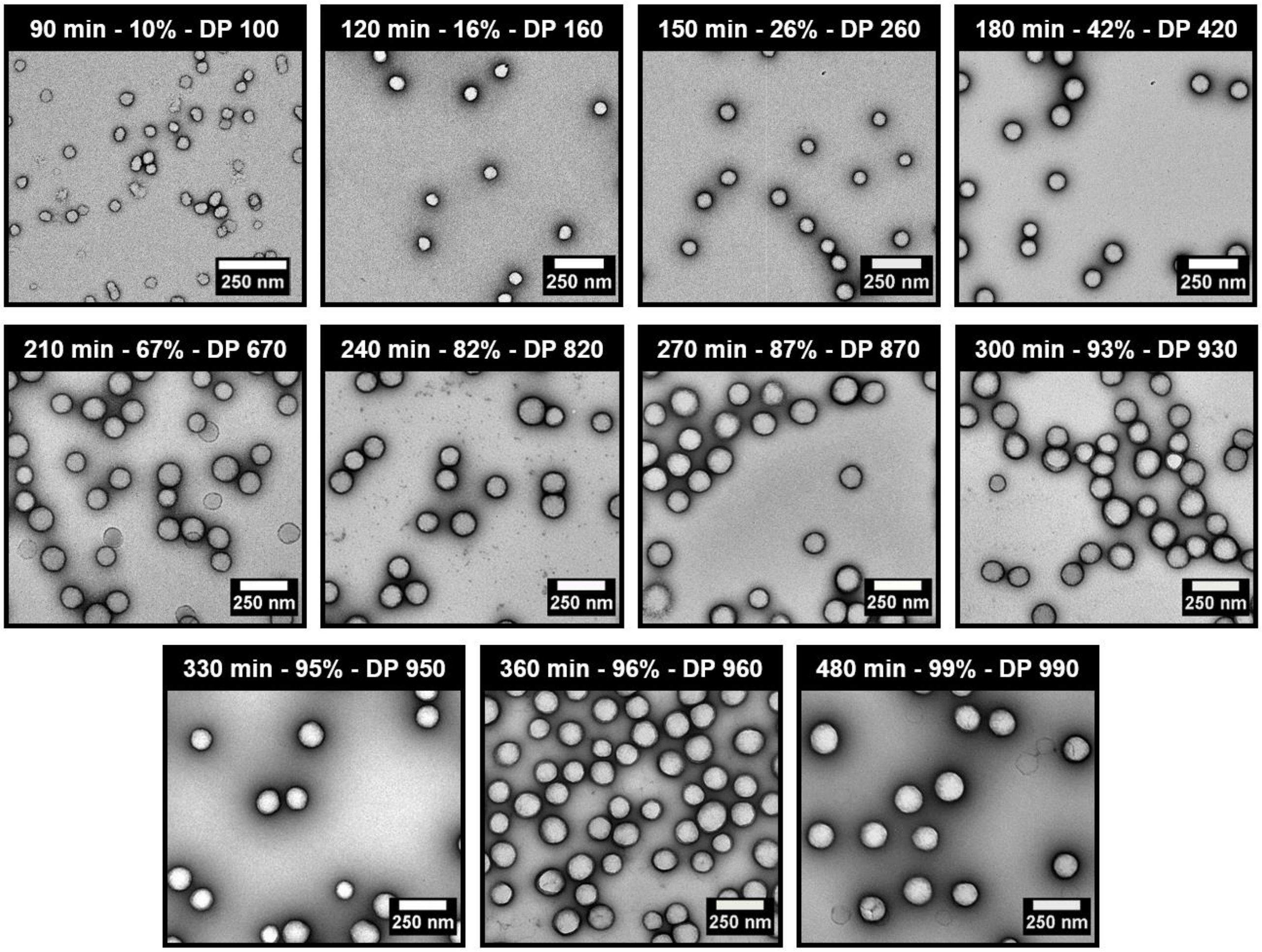


**Figure S7.** Representative TEM images recorded for $GO_{34}$-$H_y$ spherical nanoparticles obtained during kinetic studies for the synthesis of $GO_{34}$-$H_{1000}$ diblock copolymer spheres *via* RAFT aqueous dispersion polymerization of HPMA at 37 °C when targeting 10% w/w solids (using a 0.75% w/w uranyl formate stain). The polymerization time, instantaneous HPMA conversion and corresponding PHPMA DP are indicated in each case.

**Table S2.** Summary of monomer conversions, PHPMA DPs, SEC data, DLS data and morphology assignments by TEM analysis for intermediate $GO_{34}$-$H_y$ spherical nanoparticles formed during kinetic studies for the synthesis of $GO_{34}$-$H_{1000}$ diblock copolymer spheres *via* RAFT aqueous dispersion polymerization of HPMA at 37 °C targeting 10% w/w solids.

| Time (min) | HPMA conversion$^a$ (%) | PHPMA DP$^b$ | $M_{n,\,SEC}$$^c$ (kg mol$^{-1}$) | $M_w/M_n$$^c$ | $D_h$$^d$ (nm) | PD$^d$ | Derived count rate$^d$ (kcps) | Morphology$^e$ |
|---|---|---|---|---|---|---|---|---|
| 0 | 0 | 0 | 15.8 | 1.16 | - | - | 0 | - |
| 30 | 4 | 40 | 18.9 | 1.20 | - | - | 94 | *Chains* |
| 60 | 5 | 50 | 22.7 | 1.45 | - | - | 370 | *Chains* |
| 90 | 10 | 100 | 31.1 | 2.00 | 56 ± 1 | 0.07 ± 0.03 | 2,750 | Spheres |
| 120 | 16 | 160 | 47.3 | 2.96 | 74 ± 2 | 0.04 ± 0.03 | 11,300 | Spheres |
| 150 | 26 | 260 | 68.2 | 3.76 | 90 ± 1 | 0.01 ± 0.01 | 39,600 | Spheres |
| 180 | 42 | 420 | 102.5 | 4.00 | 109 ± 1 | 0.02 ± 0.02 | 104,400 | Spheres |
| 210 | 67 | 670 | 135.8 | 3.74 | 124 ± 1 | 0.01 ± 0.01 | 186,700 | Spheres |
| 240 | 82 | 820 | 159.9 | 3.38 | 130 ± 1 | 0.02 ± 0.02 | 222,000 | Spheres |
| 270 | 87 | 870 | 162.2 | 3.32 | 135 ± 2 | 0.04 ± 0.02 | 239,800 | Spheres |
| 300 | 93 | 930 | 166.3 | 3.26 | 136 ± 1 | 0.02 ± 0.01 | 239,700 | Spheres |
| 330 | 95 | 950 | 167.4 | 3.19 | 137 ± 1 | 0.02 ± 0.02 | 240,500 | Spheres |
| 360 | 96 | 960 | 171.4 | 3.18 | 137 ± 2 | 0.01 ± 0.01 | 245,700 | Spheres |
| 480 | 99 | 990 | 172.6 | 3.14 | 140 ± 2 | 0.01 ± 0.01 | 234,000 | Spheres |

$^a$Monomer conversion calculated from $^1$H NMR spectroscopy in $CD_3OD$. $^b$Calculated from the corresponding HPMA conversion. $^c$$M_n$ and $M_w/M_n$ data calculated by SEC RI analysis relative to a series of PMMA calibration standards (DMF + 10 mM LiBr eluent). $^d$$D_h$, polydispersities and derived count rate determined by DLS analysis. $^e$Morphologies assigned on the basis of TEM images, using a 0.75% w/w uranyl formate stain.

## Supplementary Characterization Data for $AGO_{34}$-$H_y$ Diblock Copolymer Spheres

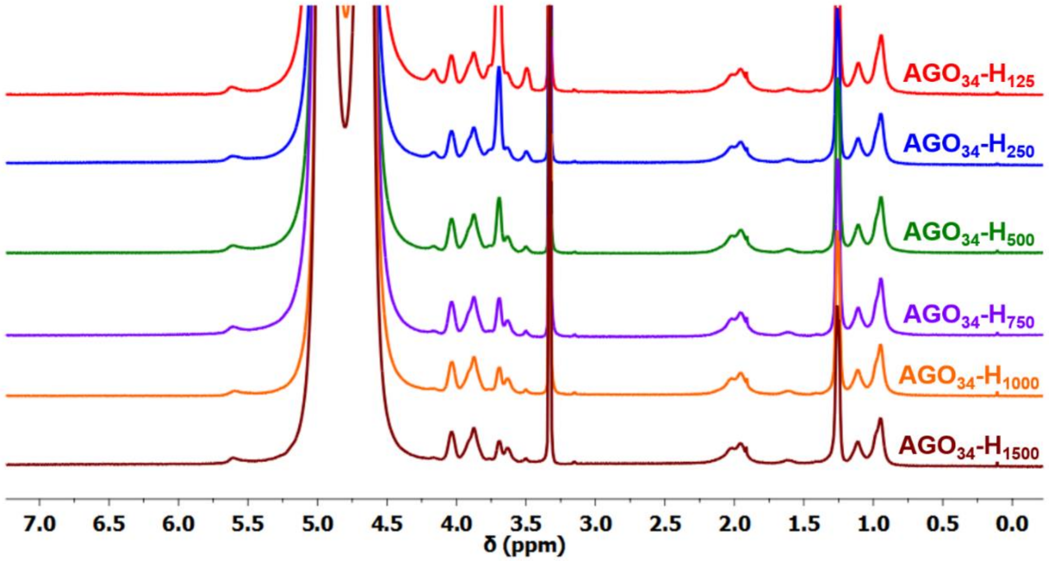


**Figure S8.** Stacked $^{1}$H NMR spectra ($CD_3OD$) recorded for a series of molecularly-dissolved $AGO_{34}$-$H_y$ diblock copolymers (where y = 125, red line; y = 250, blue line; y = 500, green line; y = 750, purple line; y = 1000, orange line; and y = 1500, burgundy line).

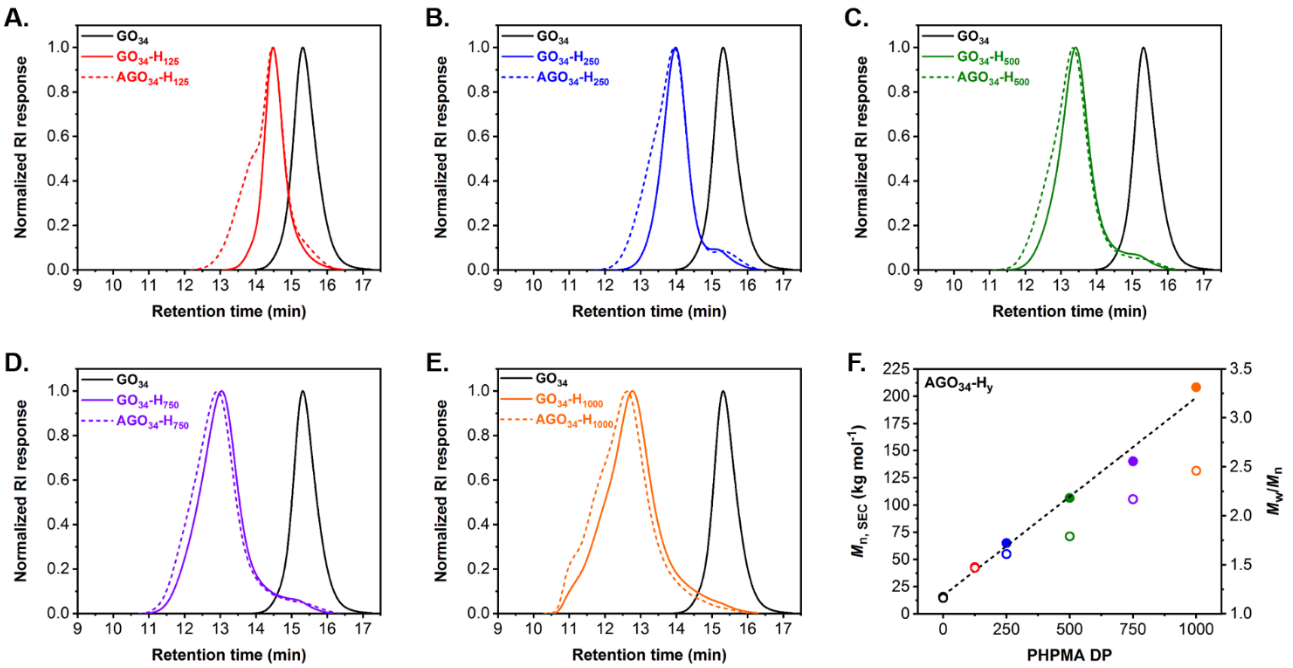


**Figure S9.** Normalized SEC curves (RI detector) recorded for the $GO_{34}$ precursor (black line) and a series of $GO_{34}$-$H_y$ (solid lines) and $AGO_{34}$-$H_y$ (dashed lines) diblock copolymers, where (A) y = 125, red lines; (B) y = 250, blue lines; (C) y = 500, green lines; (D) y = 750, purple lines; and (E) y = 1000, orange lines, (DMF + 10 mM LiBr eluent). (F) Evolution of $M_n$ (filled circles) and $M_w/M_n$ (empty circles) with increasing target PHPMA DP for a series of $AGO_{34}$-$H_y$ diblock copolymers, as calculated from SEC RI analysis using a series of PMMA calibration standards (DMF + 10 mM LiBr eluent).

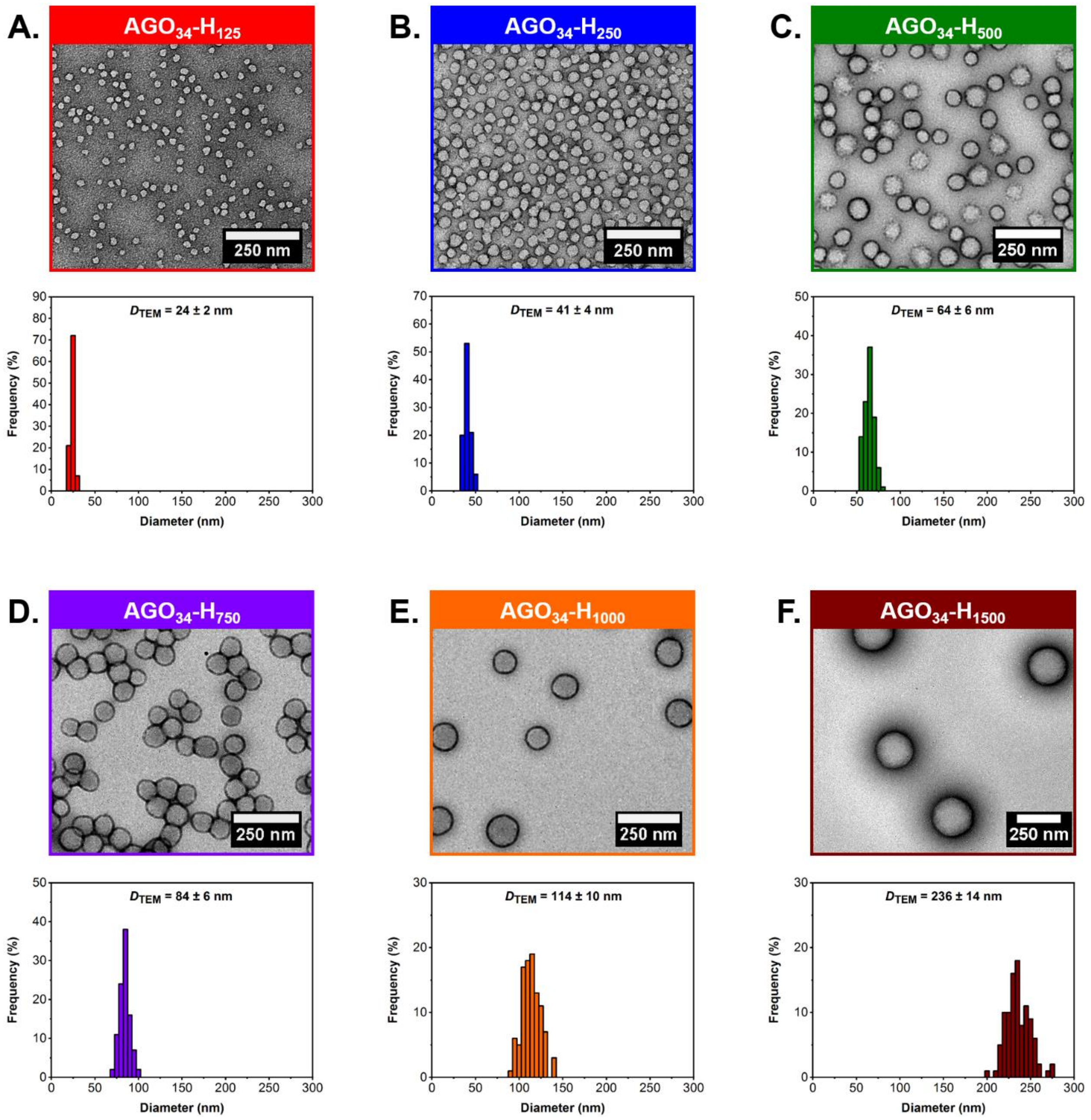


**Figure S10.** Representative TEM images recorded for $AGO_{34}$-$H_y$ diblock copolymer spheres, where (A) y = 125; (B) y = 250; (C) y = 500; (D) y = 750; (E) y = 1000; and (F) y = 1500, using a 0.75% w/w uranyl formate stain, and corresponding size distribution histograms, with the mean diameter ($D_{TEM}$), determined by analyzing at least 100 nanoparticles in each case.

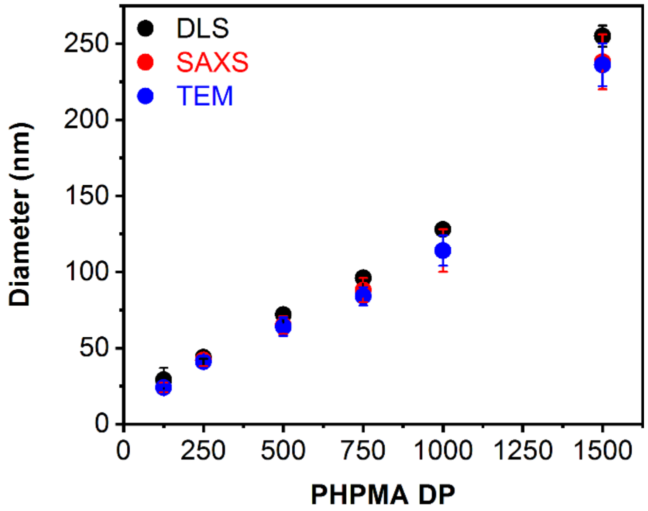


**Figure S11.** Comparison of mean diameters for a series of $AGO_{34}$-$H_y$ diblock copolymer spheres determined by DLS (black circles), SAXS (red circles) and TEM analysis (blue circles).

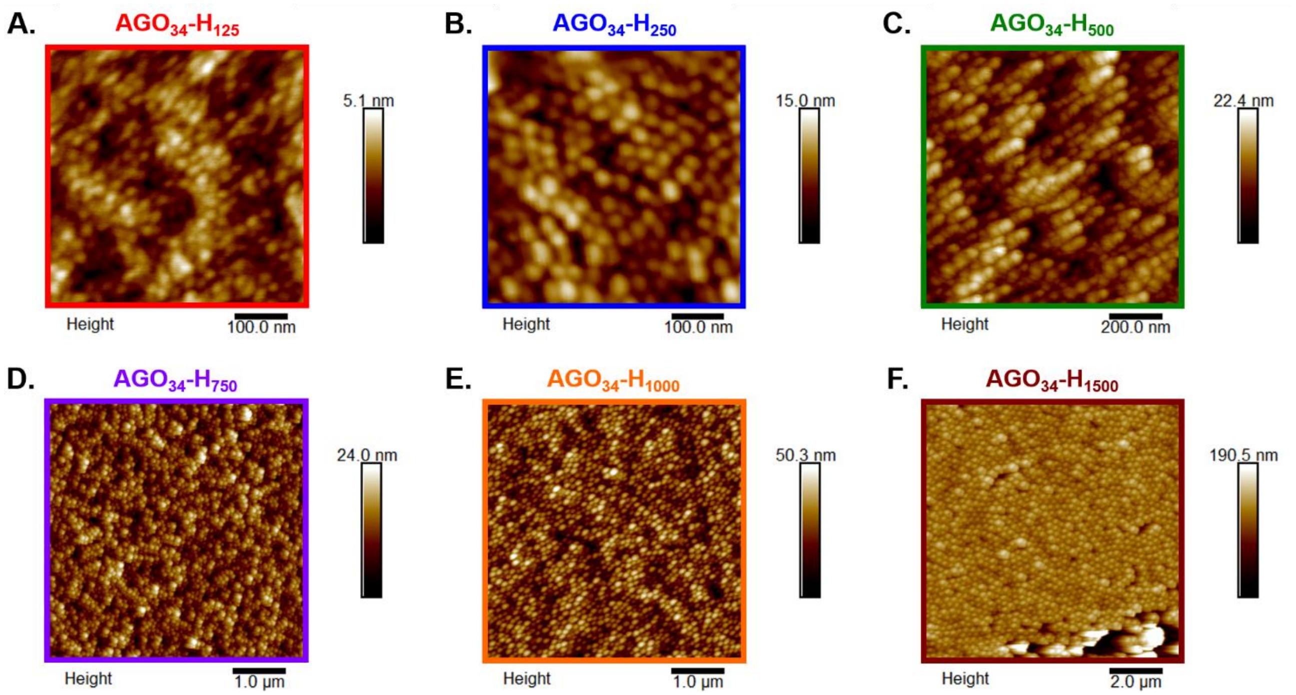


**Figure S12.** Representative AFM images recorded for a series of dried $AGO_{34}$-$H_y$ diblock copolymer spheres, where (A) y = 125; (B) y = 250; (C) y = 500; (D) y = 750; (E) y = 1000; and (F) y = 1500.

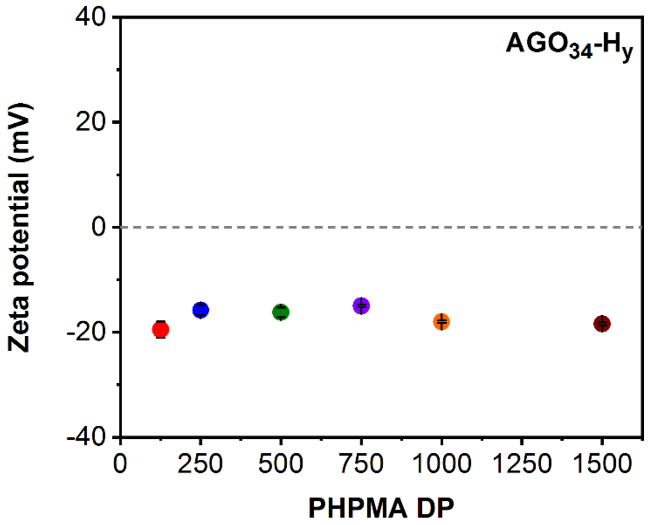


**Figure S13.** Variation in zeta potential with increasing target PHPMA DP for a series of AGO$_{34}$-H$_y$ diblock copolymer spheres, as determined by aqueous electrophoresis analysis in 1 mM KCl at pH 6.8.

**Table S3.** Summary of SEC data, DLS data, zeta potentials, SAXS and TEM mean diameters for a series of aldehyde-functional AGO$_{34}$-H$_y$ diblock copolymer spheres.

| **Copolymer** | **$M_{n, SEC}$[a] (kg mol$^{-1}$)** | **$M_w/M_n$[a]** | **$D_h$[c] (nm)** | **PD[c]** | **ζ-Potential[d] (mV) @ pH 6.8** | **$D_{SAXS}$[e] (nm)** | **$D_{TEM}$[f] (nm)** |
|---|---|---|---|---|---|---|---|
| **AGO$_{34}$-H$_{125}$** | 43.2 | 1.47 | 29 ± 8 | 0.07 ± 0.02 | - 19.5 ± 1.6 | 24 ± 3 | 24 ± 2 |
| **AGO$_{34}$-H$_{250}$** | 65.2 | 1.61 | 44 ± 1 | 0.03 ± 0.01 | - 15.8 ± 1.0 | 42 ± 4 | 41 ± 4 |
| **AGO$_{34}$-H$_{500}$** | 106.5 | 1.79 | 72 ± 2 | 0.08 ± 0.01 | - 16.2 ± 0.9 | 65 ± 6 | 64 ± 6 |
| **AGO$_{34}$-H$_{750}$** | 140.3 | 2.17 | 96 ± 2 | 0.02 ± 0.01 | - 15.0 ± 0.3 | 88 ± 8 | 84 ± 6 |
| **AGO$_{34}$-H$_{1000}$** | 208.3 | 2.46 | 128 ± 2 | 0.03 ± 0.02 | - 18.0 ± 0.2 | 114 ± 14 | 114 ± 10 |
| **AGO$_{34}$-H$_{1500}$** | -[b] | -[b] | 255 ± 7 | 0.02 ± 0.01 | - 18.4 ± 0.3 | 238 ± 18 | 236 ± 14 |

[a]$M_n$ and $M_w/M_n$ data calculated by SEC RI analysis relative to a series of PMMA calibration standards (DMF + 10 mM LiBr eluent). [b]Poor solubility in SEC eluent. [c]$D_h$ and polydispersities determined by DLS analysis. [d]Zeta potentials determined by aqueous electrophoresis conducted in 1 mM KCl at pH 6.8. [e]$D_{SAXS}$ determined by fitting the corrsponding SAXS patterns. [f]$D_{TEM}$ determined from TEM images by analyzing at least 100 nanoparticles in each case.

## Supplementary Characterization Data for PGEO5MA, PAGEO5MA, PDAGEO5MA and (A)$GO_{34}$-$H_y$-Decorated PDAGEO5MA Brushes

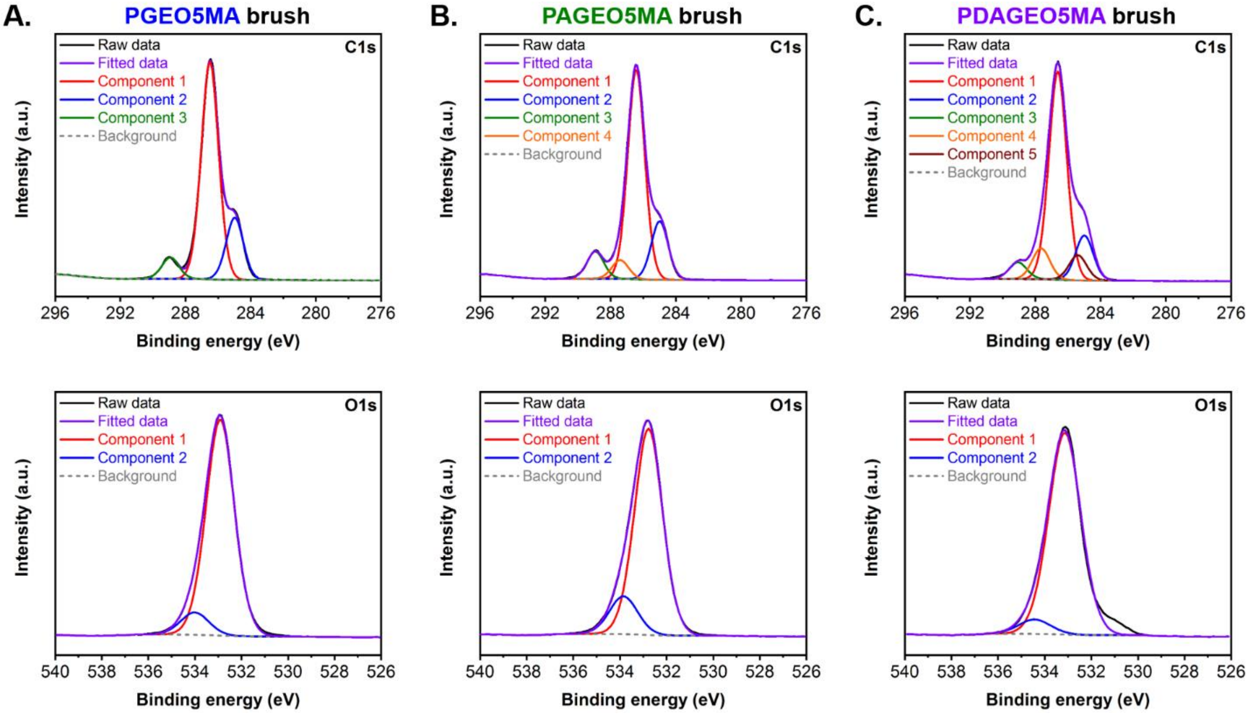


**Figure S14.** High-resolution C1s (top) and O1s (bottom) spectra recorded for (A) PGEO5MA, (B) PAGEO5MA, and (C) PDAGEO5MA brushes (black lines). The fitted data (purple lines), derived individual components (red, blue, green, orange and burgundy lines) and subtracted background (grey dashed lines) are shown in each case.

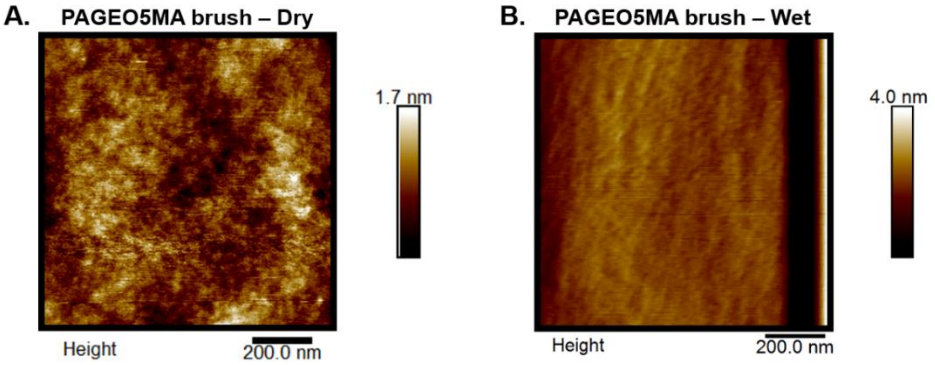


**Figure S15.** Representative AFM images recorded for (A) a dry PAGEO5MA brush (mean dry brush thickness = 111 nm) and (B) the corresponding hydrated PAGEO5MA brush.

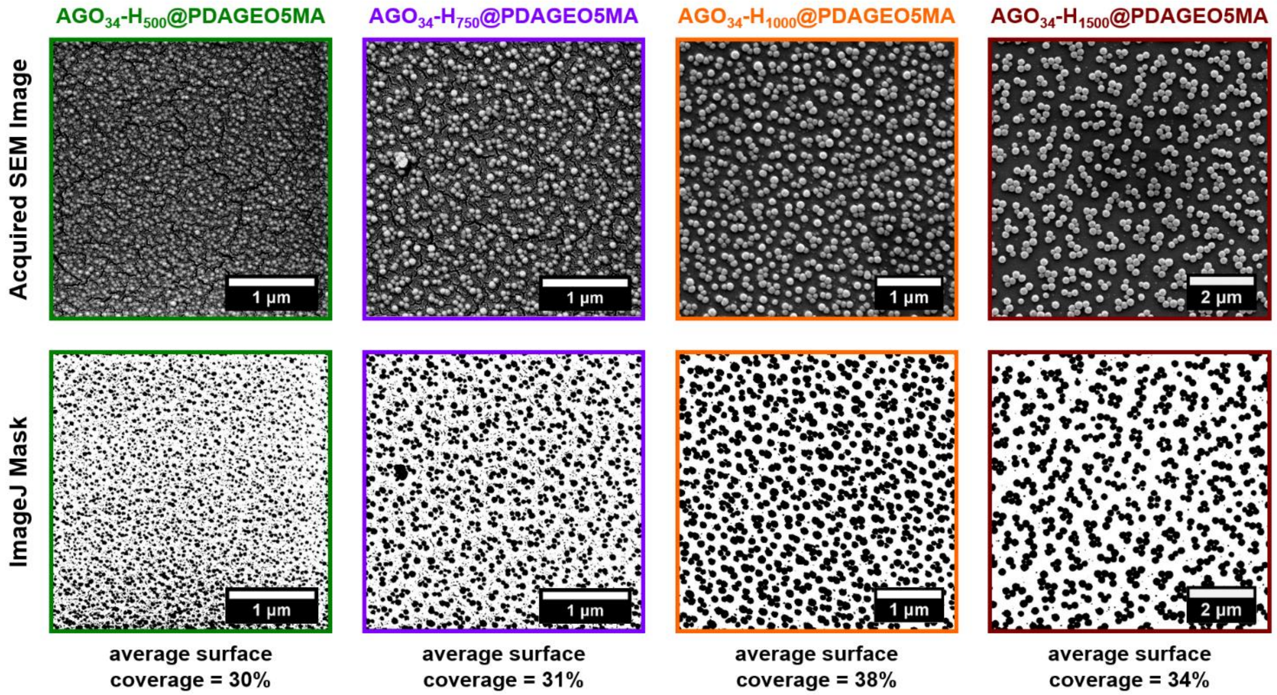


**Figure S16.** Representative SEM images, corresponding mask images generated using ImageJ software and mean surface coverages (%) for a series of nanoparticle-decorated $AGO_{34}$-$H_y$@PDAGEO5MA surfaces (where y = 500-1500).

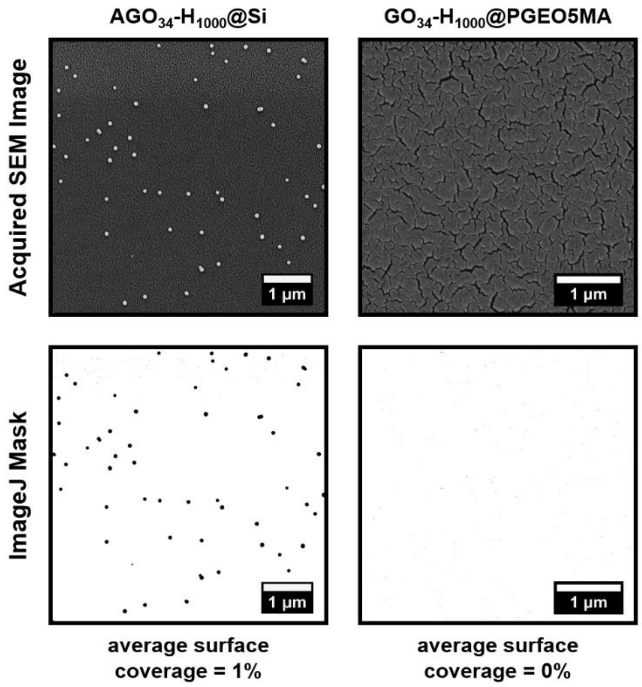


**Figure S17.** Representative SEM images, corresponding mask images generated using ImageJ software and mean surface coverages (%) for $AGO_{34}$-$H_{1000}$@Si and $GO_{34}$-$H_{1000}$@PGEO5MA control samples.

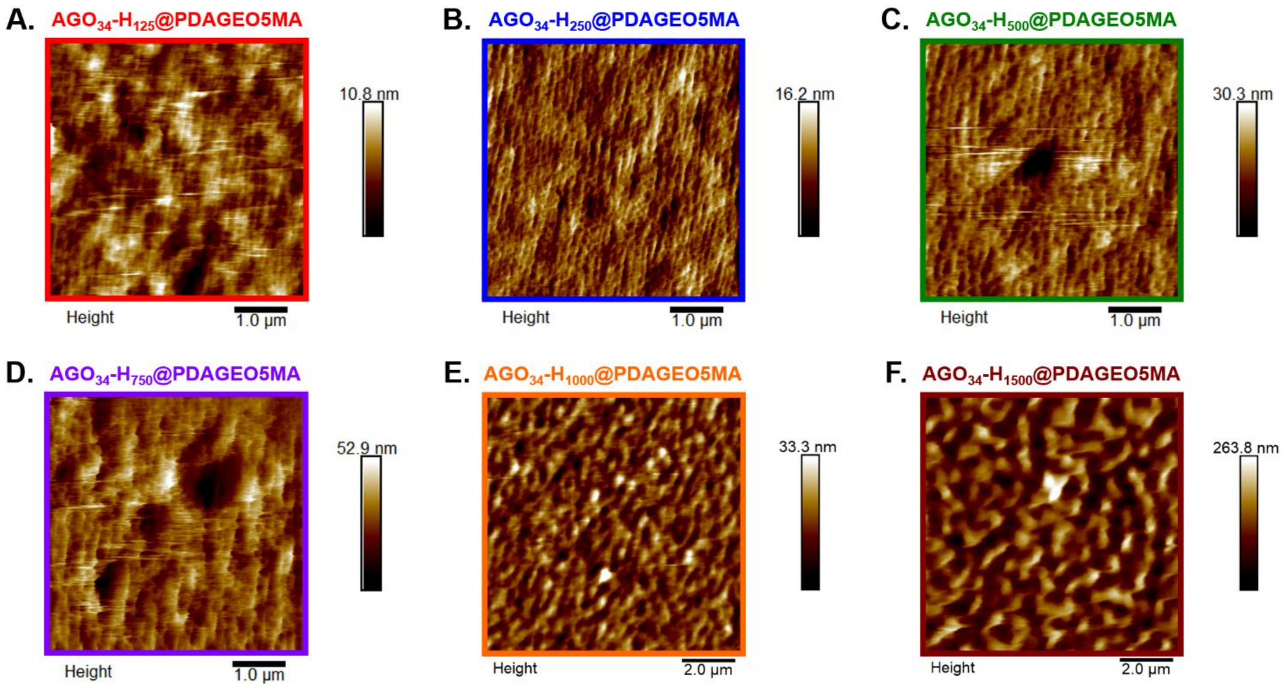


**Figure S18.** Representative AFM images recorded for a series of hydrated $AGO_{34}$-$H_y$@PDAGEO5MA surfaces, where (A) y = 125; (B) y = 250; (C) y = 500; (D) y = 750; (E) y = 1000; and (F) y = 1500.

**Table S4.** Summary of dry thicknesses for PGEO5MA, PAGEO5MA and PDAGEO5MA brushes and nanoparticle-decorated $AGO_{34}$-$H_y$@PDAGEO5MA surfaces, along with degrees of diamine functionalization (%), as determined by ellipsometry. Mean values are also provided where appropriate.

| **Entry** | **PGEO5MA dry brush thickness (nm)** | **PAGEO5MA dry brush thickness (nm)** | **PDAGEO5MA dry brush thickness (nm)** | **Functionalization w. diamine (%)** | **Nanoparticle modifier** | **$AGO_{34}$-$H_y$ @PDAGEO5MA dry thickness (nm)** |
|---|---|---|---|---|---|---|
| **1** | 120.7 | 108.0 | 127.6 | 40.4 | **$AGO_{34}$-$H_{125}$** | 142.6 |
| **2** | 111.6 | 105.5 | 123.9 | 39.1 | **$AGO_{34}$-$H_{250}$** | 161.5 |
| **3** | 123.9 | 113.8 | 130.9 | 33.6 | **$AGO_{34}$-$H_{500}$** | 174.2 |
| **4** | 127.9 | 117.9 | 137.0 | 36.4 | **$AGO_{34}$-$H_{750}$** | 175.5 |
| **5** | 118.4 | 105.9 | 119.2 | 28.1 | **$AGO_{34}$-$H_{1000}$** | 160.1 |
| **6** | 125.8 | 113.5 | 132.9 | 38.2 | **$AGO_{34}$-$H_{1500}$** | 232.3 |
| **Mean** | 121.4 | 110.8 | 128.6 | 36.0 | - | - |

# References


1. Brotherton, E. E.; Jesson, C. P.; Warren, N. J.; Smallridge, M. J.; Armes, S. P. New Aldehyde-Functional Methacrylic Water-Soluble Polymers. *Angew. Chem. Int. Ed.* **2021**, *60*, 12032-12037.
2. Jones, E. R.; Semsarilar, M.; Blanazs, A.; Armes, S. P. Efficient Synthesis of Amine-Functional Diblock Copolymer Nanoparticles via RAFT Dispersion Polymerization of Benzyl Methacrylate in Alcoholic Media. *Macromolecules* **2012**, *45*, 5091-5098.
3. Penfold, N. J. W.; Lovett, J. R.; Warren, N. J.; Verstraete, P.; Smets, J.; Armes, S. P. pH-Responsive non-ionic diblock copolymers: protonation of a morpholine end-group induces an order–order transition. *Polym. Chem.* **2016**, *7*, 79-88.
4. Ilavsky, J.; Jemian, P. R. Irena: tool suite for modeling and analysis of small-angle scattering. *J. Appl. Crystallogr.* **2009**, *42*, 347-353.
5. Pedersen, J. S.; Gerstenberg, M. C. Scattering Form Factor of Block Copolymer Micelles. *Macromolecules* **1996**, *29*, 1363-1365.
6. Brotherton, E. E.; Smallridge, M. J.; Armes, S. P. Aldehyde-Functional Diblock Copolymer Nano-objects via RAFT Aqueous Dispersion Polymerization. *Biomacromolecules* **2021**, *22*, 5382-5389.
7. Johnson, E. C.; Willott, J. D.; de Vos, W. M.; Wanless, E. J.; Webber, G. B. Interplay of Composition, pH, and Temperature on the Conformation of Multi-stimulus-responsive Copolymer Brushes: Comparison of Experiment and Theory. *Langmuir* **2020**, *36*, 5765-5777.
8. Brotherton, E. E.; Johnson, E. C.; Smallridge, M. J.; Hammond, D. B.; Leggett, G. J.; Armes, S. P. Hydrophilic Aldehyde-Functional Polymer Brushes: Synthesis, Characterization, and Potential Bioapplications. *Macromolecules* **2023**, *56*, 2070-2080.
9. Genzer, J. In Silico Polymerization: Computer Simulation of Controlled Radical Polymerization in Bulk and on Flat Surfaces. *Macromolecules* **2006**, *39*, 7157-7169.
10. Gorman, C. B.; Petrie, R. J.; Genzer, J. Effect of Substrate Geometry on Polymer Molecular Weight and Polydispersity during Surface-Initiated Polymerization. *Macromolecules* **2008**, *41*, 4856-4865.
11. Willott, J. D.; Murdoch, T. J.; Webber, G. B.; Wanless, E. J. Nature of the Specific Anion Response of a Hydrophobic Weak Polyelectrolyte Brush Revealed by AFM Force Measurements. *Macromolecules* **2016**, *49*, 2327-2338.

12. Murdoch, T. J.; Humphreys, B. A.; Willott, J. D.; Prescott, S. W.; Nelson, A.; Webber, G. B.; Wanless, E. J. Enhanced specific ion effects in ethylene glycol-based thermoresponsive polymer brushes. *J. Colloid Interface Sci.* **2017**, *490*, 869-878.
13. Murdoch, T. J.; Humphreys, B. A.; Johnson, E. C.; Prescott, S. W.; Nelson, A.; Wanless, E. J.; Webber, G. B. The role of copolymer composition on the specific ion and thermo-response of ethylene glycol-based brushes. *Polymer* **2018**, *138*, 229-241.
